# Quantitative measurement of the requirement of diverse protein degradation pathways in MHC class I peptide presentation

**DOI:** 10.1126/sciadv.ade7890

**Published:** 2023-06-23

**Authors:** Jennifer L. Mamrosh, David J. Sherman, Joseph R. Cohen, James A. Johnston, Marisa K. Joubert, Jing Li, J. Russell Lipford, Brett Lomenick, Annie Moradian, Siddharth Prabhu, Michael J. Sweredoski, Bryan Vander Lugt, Rati Verma, Raymond J. Deshaies

**Affiliations:** ^1^ Division of Biology and Biological Engineering, California Institute of Technology, Pasadena, CA 91125, USA.; ^2^ Amgen Research, Thousand Oaks, CA 91320, USA.; ^3^ Process Development, Amgen Inc., Thousand Oaks, CA 91320, USA.; ^4^ Proteome Exploration Laboratory, California Institute of Technology, Pasadena, CA 91125, USA.; ^5^ Amgen Research, South San Francisco, CA 94080, USA.

## Abstract

Peptides from degradation of intracellular proteins are continuously displayed by major histocompatibility complex (MHC) class I. To better understand origins of these peptides, we performed a comprehensive census of the class I peptide repertoire in the presence and absence of ubiquitin-proteasome system (UPS) activity upon developing optimized methodology to enrich for and quantify these peptides. Whereas most class I peptides are dependent on the UPS for their generation, a surprising 30%, enriched in peptides of mitochondrial origin, appears independent of the UPS. A further ~10% of peptides were found to be dependent on the proteasome but independent of ubiquitination for their generation. Notably, clinically achievable partial inhibition of the proteasome resulted in display of atypical peptides. Our results suggest that generation of MHC class I•peptide complexes is more complex than previously recognized, with UPS-dependent and UPS-independent components; paradoxically, alternative protein degradation pathways also generate class I peptides when canonical pathways are impaired.

## Teaser

The contribution of protein degradation pathways to MHC Class I peptide generation was quantified by mass spectrometry.

## INTRODUCTION

To survive, organisms must be able to detect threats in their environment. While all multicellular organisms have an innate immune system broadly surveying for common pathogens, evolution of an adaptive immune system allowed for specific and lasting responses to wider threats. Central to adaptive immunity is the display of antigens. All nucleated cells in jawed vertebrates display on their cell surface peptide antigens derived from intracellular proteins. This represents the current state of cells to the immune system and allows for the detection of intracellular infection by bacteria and viruses. Peptides typically nine amino acids in length, generated from intracellular proteins, are presented in a noncovalent complex with the plasma membrane protein major histocompatibility complex (MHC) class I. These peptides, largely originating as longer peptides generated by proteasomal degradation, often are subject to additional trimming by proteases in the cytoplasm and endoplasmic reticulum (ER) before being loaded onto MHC class I (*1*). It is generally assumed that ER aminopeptidases ERAP1/2 generate the N-terminal amino acid of MHC class I peptides, while proteasomes generate the C-terminal amino acid.

Which peptides are displayed by MHC class I is dependent on binding preferences of hypervariable class I genes (*HLA-A,-B,-C*) (*2*). Far less is understood regarding how these peptides are generated from intracellular proteins by protein degradation pathways. It was initially reported that the bulk of MHC class I peptide generation is dependent on ubiquitination and proteasomal degradation (*3*, *4*). However, the necessity of ubiquitination (*5*, *6*) and proteasomal degradation (*7*) has been questioned, particularly in certain contexts such as ubiquitin-independent presentation of viral peptides (*8*) and in individuals with MHC class I alleles more likely to present proteasome-independent peptides (*9*). Our goal here was to quantify the role of the ubiquitin-proteasome system (UPS) in endogenous MHC class I peptide presentation more conclusively at the level of individual peptides from proteins in the canonical proteome, although peptide generation from the noncanonical proteome has also been reported (*10*).

Our work, which represents the first application of quantitative mass spectrometry to estimate the contribution of the UPS to MHC class I peptide generation, allowed us to determine characteristics of proteins dependent on UPS pathways for generation of MHC class I peptides, as well as to determine whether specific components of these pathways (*11*) play an outsized role in peptide generation. Technical optimizations were made to enable high-throughput and quantitative MHC class I peptide mass spectrometry following chemical inhibition of protein degradation pathways, allowing us to identify peptides presented by MHC class I that are dependent on these pathways. Most experiments were performed in immortalized B lymphoblasts, which abundantly present MHC class I peptides while generally not cross-presenting peptides typically associated with MHC class II [i.e., peptides from extracellular or endolysosomal pathway proteins (*12*)]. Initially, we inhibited ubiquitination and proteasomal degradation, and observed that, in line with findings from initial reports (*3*, *4*) but contrary to some more recent reports (*5*–*7*, *9*), UPS pathways are largely required for MHC class I peptide presentation. However, we also identified evidence for presentation of atypical peptides when these canonical UPS pathways were inhibited, as well as a surprising number of proteasome-dependent substrates less dependent on ubiquitination. This prompted us to consider the relative contributions of autophagy as well as proteasome-associated factors such as p97/VCP to MHC class I peptide generation. Our experiments demonstrate that these protein degradation pathways generate specific subsets of MHC class I peptides, and also suggest that MHC class I peptide display is relatively robust in the face of environmental insult to protein degradation pathways. Remarkably, partial inhibition of the proteasome also induces the presentation of a small number of atypical peptides, suggesting that proteasome inhibition may have clinical utility in cancer immunotherapy contexts.

## RESULTS

### MHC class I peptide mass spectrometry is improved by methodology for more accurate data normalization and removal of “background peptides”

**Fig. 1.**
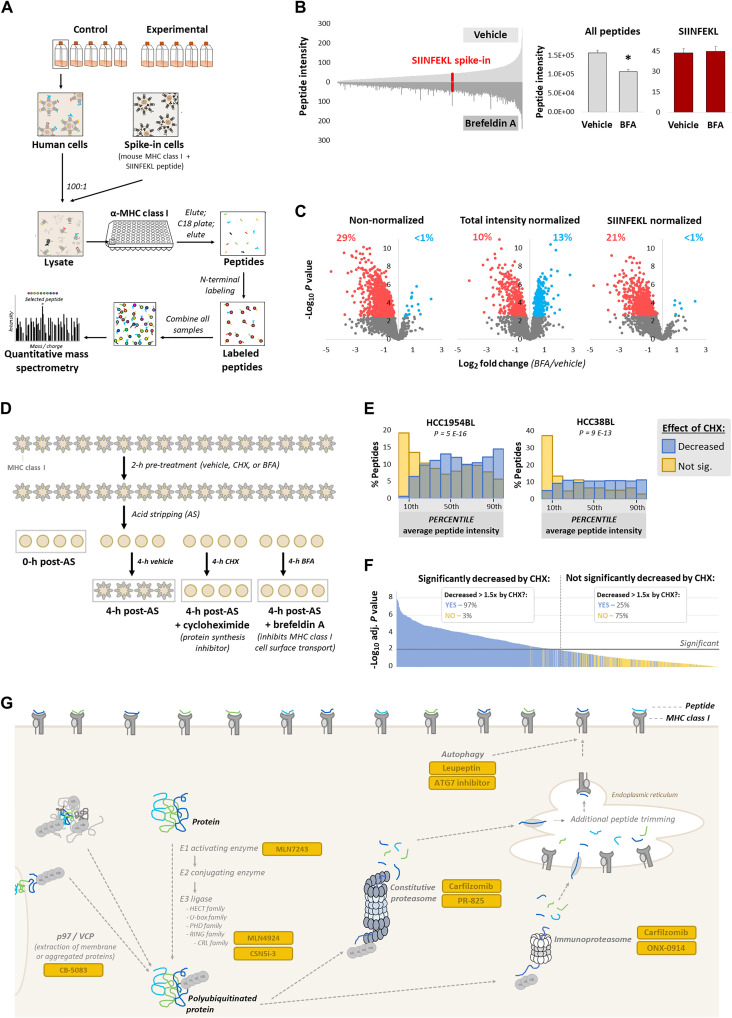
Improved methodology for MHC class I peptide mass spectrometry. (**A**) Experimental design for mass spectrometry quantification of MHC class I peptides. (**B**) Left: Average mass spectrometry individual peptide intensity from vehicle-treated (top) and brefeldin A (BFA)–treated (bottom) cells. SIINFEKL spike-in peptide is in red. Right: Summed total peptide intensity and SIINFEKL spike-in peptide intensity. *P < 0.01 by *t* test. (**C**) Changes in MHC class I peptide presentation upon BFA treatment (red indicates significantly decreased; blue indicates significantly increased). Data are presented as nonnormalized, total intensity normalized (each sample normalized to its summed peptide intensity), and normalized to the spike-in SIINFEKL peptide. Percent significant (increased and decreased) are marked above plots. (**D**) Experimental design to block MHC class I peptide presentation; expected impact of treatments is depicted. (**E**) The average peptide intensity at 4 hours after acid stripping, representing constitutive antigen presentation, was determined for two groups: peptides significantly decreasing in response to cycloheximide (CHX) treatment (blue), and those not significantly decreasing (yellow). Average peptide intensity percentiles, compared to all peptides identified, were determined and presented as histograms (overlap is indicated by green); *P* value was determined by *t* test. (**F**) The following groups were compared: peptides significantly decreasing in response to CHX treatment, and those not. Significance for each peptide is plotted. Another comparison using this dataset was performed, using just the first two replicates of the CHX treatment group. Peptides decreased greater than 1.5-fold in response to CHX in both replicates versus vehicle are plotted in blue, and all others plotted in yellow. In the legend, “Decreased > 1.5× by CHX” means for both replicates. (**G**) Protein degradation pathways inhibited in our studies.

We purified MHC class I peptides as previously described (*13*) and subsequently labeled samples on their N termini with mass isomers to enable relative quantification by multiplexing in a single mass spectrometry run. Additionally, we developed a “spike-in standard” consisting of mouse cells expressing MHC class I allele H-2K^b^ cross-presenting a defined peptide (SIINFEKL) and mixed this lysate at a 1:100 ratio based on cell number with our human cell lysates. This mouse MHC class I•SIINFEKL standard was then copurified with human MHC class I complexes, using an H-2K^b^•SIINFEKL complex antibody mixed with a pan-human MHC class I antibody at a 1:100 ratio (Fig. 1A). The spike-in standard enabled more accurate normalization to correct for potential sample loss during the peptide purification steps. Additionally, it allowed for normalization when experimental treatment altered overall MHC class I peptide presentation. This in particular is critical if one wishes to test a condition (e.g., proteasome inhibition) that has the potential to reduce presentation. To demonstrate the value of this normalization, B lymphoblasts were treated with the secretion inhibitor brefeldin A for 16 hours, which inhibits transport of MHC class I to the cell surface (*14*). MHC class I peptides quantified by mass spectrometry were as expected in size (fig. S1A). Whereas brefeldin A reduced overall MHC class I peptide presentation as evidenced by total peptide intensity measured by mass spectrometry, levels of the spike-in standard were not affected (Fig. 1B). Statistical analysis of mass spectrometry data by widely used data normalization methods like total intensity normalization, however, obscured this treatment-induced decrease in total MHC class I presentation (Fig. 1C). Normalization to the spike-in standard avoided this artifact while also correcting for any potential sample loss during processing steps.

Ultimately, we aimed to perform experiments on cells treated with protein degradation pathway inhibitors for just a few hours due to the toxicity of inhibiting core pathways. Removal of preexisting MHC class I complexes by brief incubation of cells in mild acid solution (“acid stripping”) (*15*, *16*) resulted in >90% reduction in cell surface class I complexes, with substantial recovery by 4 hours (fig. S1B). Cells were pretreated for 2 hours with either brefeldin A, the translation inhibitor cycloheximide to block production of new proteins and MHC class I complexes, or vehicle, and then acid-stripped. Cells were immediately collected following acid stripping, or else cultured again for 4 hours with inhibitors (Fig. 1D). We observed that total peptide intensity measured by mass spectrometry was markedly reduced following acid stripping or upon recovery in cycloheximide; this reduction was lesser upon recovery in brefeldin A (fig. S1C), since loaded MHC class I complexes can potentially be purified from the ER even if not transported to the cell surface (*17*). Nevertheless, total peptide abundance was not as reduced as we expected even following acid stripping or cycloheximide treatment. We considered that this might be a limitation of mass spectrometry, such as background quantification. Additionally, the preference of mass spectrometry for quantifying abundant peptides (*18*) likely biased toward selection of MHC class I–bound peptides that decreased less in response to treatment. To better understand these “background peptides,” we specifically considered the effects of cycloheximide treatment, which was comparable to effects seen immediately upon acid stripping (fig. S1D). We observed that peptides that did not significantly decrease in response to cycloheximide were lower in intensity, leading us to suspect that many peptides near the limit of detection by mass spectrometry suffer from background quantification or are too variable to detect a significant decrease (Fig. 1E). For most subsequent mass spectrometry experiments, we included two cycloheximide-treated replicates and excluded from further analysis peptides not decreasing greater than 1.5-fold in response to cycloheximide in both replicates. This filter removed from further consideration a large number of background peptides that were not statistically significant (Fig. 1F), without requiring the three or more replicates needed for a cutoff based on statistical significance. We then sought to apply this optimized methodology for quantitative MHC class I peptide mass spectrometry to determine the role of diverse protein degradation pathways in MHC class I peptide generation (Fig. 1G).

### Inhibition of ubiquitination and proteasomal degradation results in a net decrease in MHC class I peptide presentation, yet paradoxical increases in certain peptides

In four B lymphoblast cell lines with largely distinct MHC class I alleles (fig. S1E), we planned to inhibit ubiquitination with the ubiquitin activating enzyme E1 inhibitor MLN7243 and the proteasome with carfilzomib. We determined a dose of MLN7243 (500 nM) resulting in disappearance of most polyubiquitinated proteins with 4 hours of pretreatment (fig. S2A), and a dose of carfilzomib (1 μM) resulting in near-complete proteasome inhibition with 1 hour of pretreatment (fig. S2, B and C). Proteasomal degradation of known substrates was inhibited with this dose of carfilzomib (fig. S2D). We did not observe significant translational inhibition following MLN7243 or carfilzomib pretreatment (fig. S2E). For mass spectrometry experiments, cells were pretreated with these inhibitors, preexisting MHC class I peptides were removed by acid stripping, and cells were treated again with inhibitors for 4 hours. These treatments did not affect cell viability (fig. S2F). As measured by flow cytometry (fig. S2G) and mass spectrometry (fig. S2H), inhibition of ubiquitination or proteasomal degradation reduced overall MHC class I peptide presentation.

Reduction in MHC class I peptide presentation upon inhibition of ubiquitination or proteasomal degradation was unlikely to be a nonspecific outcome of inhibitor toxicity, as we performed mass spectrometry on cells treated with similarly toxic or more toxic doses of the DNA damaging agent cisplatin (fig. S2I), and observed significant decreases in peptide presentation only at levels of cisplatin treatment substantially more toxic than that of proteasome inhibition (fig. S2J).

**Fig. 2.**
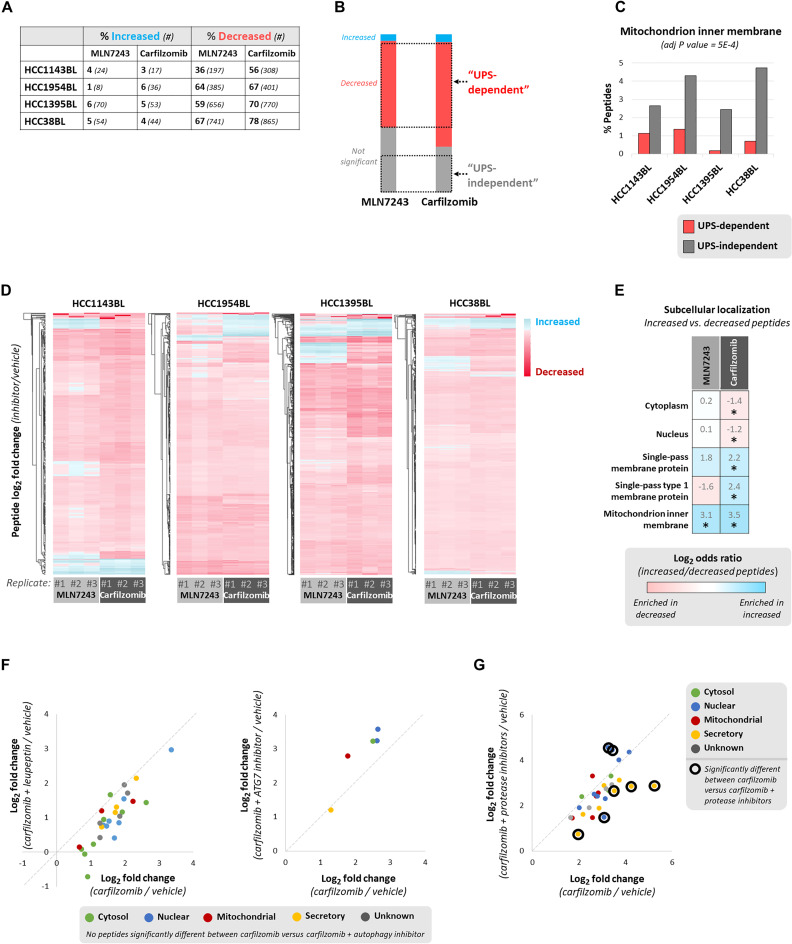
Inhibition of ubiquitination and proteasomal degradation decreases global MHC class I peptide presentation, yet paradoxically increases certain peptides. (**A**) Percent (bold) and number (italicized) of MHC class I peptides significantly increased or decreased by 4 hours of MLN7243 (500 nM; 4-hour pretreatment) or carfilzomib (1 μM; 1-hour pretreatment) treatment versus vehicle. Peptides not decreased >1.5× by cycloheximide (25 μg/ml; 2-hour pretreatment) were excluded. (**B**) Criteria for classification of UPS-dependent and UPS-independent MHC class I peptides. Bar charts represent combined data from cell lines in (A) and are proportionally accurate. (**C**) Subcellular localization for UPS-independent and UPS-dependent peptides was obtained from UniProt, and a Cochran-Mantel-Haenszel test was used to test the enrichment of subcellular localization terms across all cell lines. (**D**) Heatmap of peptides increased (blue) or decreased (red) by MLN7243 and carfilzomib treatment. Log_2_ fold change (inhibitor/vehicle) is depicted only for peptides significant in at least one treatment. (**E**) Enrichment of subcellular localization terms for peptides significantly increased versus peptides significantly decreased in cells treated with MLN7243 or carfilzomib. A Cochran-Mantel-Haenszel test determined the enrichment of subcellular localization terms across all cell lines; *Adjusted *P* < 0.05. (**F**) HCC1954BL cells were treated for 4 hours with carfilzomib (1 μM; 1-hour pretreatment) and/or leupeptin (50 μM; 1-hour pretreatment); similarly, they were treated with carfilzomib (1 μM; 1-hour pretreatment) and/or an ATG7 inhibitor (25 μM; 1-hour pretreatment). Only MHC class I peptides significantly increased by carfilzomib are depicted. (**G**) HCC1954BL cells were treated for 4 hours with carfilzomib (1 μM; 1-hour pretreatment) and/or protease inhibitors (300 μM E64 and 50 μM ALLM; 1-hour pretreatment). Only MHC class I peptides significantly increased by carfilzomib are depicted. Peptides significantly different between carfilzomib and carfilzomib and protease inhibitor treatment groups are circled.

Whereas we observed an expected decrease in MHC class I peptide presentation upon inhibition of ubiquitination or proteasomal degradation, a substantial fraction of peptides was not significantly decreased by these treatments (Fig. 2A). Some of these peptides showed a trend toward decreasing that might become statistically significant if larger sample sizes were used, although this appears unlikely to be true for most of these peptides (fig. S3A). We also considered that peptides not decreasing in response to ubiquitination or proteasome inhibition might be derived from proteins with unusually long half-lives. To address this, we obtained reported half-lives of source proteins for MHC class I peptides significantly decreased by both carfilzomib and MLN7243 treatment (“UPS-dependent”), and those not significantly decreased by either treatment (“UPS-independent”) (Fig. 2B). While the average half-lives of proteins encoding UPS-independent peptides were longer than UPS-dependent peptides, the small magnitude of this effect suggests that most peptides considered UPS-independent do not have half-lives prohibitively long for MHC class I peptide generation (fig. S3B).

We considered the possibility that MHC class I peptides presented seemingly independent of the UPS could be produced by alternative pathways. UPS-independent peptides were more likely to be derived from mitochondrial inner membrane proteins (Fig. 2C), a region of the cell inaccessible to proteasomes, and were more likely to be longer in length than the typical 9-mer (fig. S3C), suggesting that protein fragments produced by the proteasome are more optimal for MHC class I peptide display. It is possible that these peptides are generated by mitochondrial inner membrane proteases such as LonP1, ClpP, and YME1L1, which are not expected to be sensitive to MLN7243 or carfilzomib.

Unexpectedly, we also observed that some peptides were increased by MLN7243 or carfilzomib treatment (Fig. 2A), which we termed as “alternative” MHC class I peptide generation. Alternative class I peptide generation pathways could increase in activity upon inhibition of the UPS; alternatively, the activity of this pathway(s) could remain unchanged but the peptides it produces could be better able to bind MHC class I in the absence of higher-affinity canonical peptides. Significantly increased peptides often appeared to be inhibitor specific (Fig. 2D), suggesting that they were not produced by nonspecific cell stress pathways; in support of this, we also observed few increased peptides upon cisplatin treatment beyond rapidly induced proteins like HMOX1 (fig. S2J) (*19*). By comparing significantly increased versus significantly decreased peptides across all cell lines, we observed that peptides significantly decreased in response to proteasome inhibition were more likely to come from cytosolic and nuclear proteins, whereas those that increased were more likely to be from single-pass membrane proteins, particularly type 1 membrane proteins, and mitochondrial inner membrane proteins (Fig. 2E). Peptides increased by inhibition of ubiquitination were also more likely to come from mitochondrial inner membrane proteins. Therefore, we suspected that MHC class I peptides increased by proteasome inhibition, and perhaps inhibition of ubiquitination, were from proteins typically not exposed to the proteasome (e.g., mitochondrial and membrane proteins) and instead were perhaps degraded by extracytoplasmic proteases.

To test the hypothesis that MHC class I peptides increased by proteasome inhibition were produced by autophagy or other forms of lysosomal degradation, we treated cells with carfilzomib in the presence or absence of the autophagy/lysosomal inhibitor leupeptin (fig. S3D) and quantified peptide presentation by mass spectrometry. Leupeptin treatment decreased overall MHC class I peptide presentation, although less so than carfilzomib (fig. S3E), potentially due to its milder inhibition of select proteasomal activity (*20*). Leupeptin had a more marked impact on presentation of peptides, with C-terminal amino acids likely to be generated by trypsin-like enzymatic activity (fig. S3F). Similarly, we also treated cells with carfilzomib in the presence or absence of an ATG7 inhibitor (*21*) (fig. S3G), which decreased peptide presentation less significantly than leupeptin (fig. S3H). These autophagy inhibitors did not prevent the increased presentation of certain peptides observed with carfilzomib treatment (Fig. 2F). Therefore, autophagy or lysosomal degradation, at least that can be inhibited by the lysosomal enzyme inhibitor leupeptin or the ATG7 inhibitor blocking the induction of autophagy, does not appear to be the major source of MHC class I peptides increased by carfilzomib treatment.

Therefore, we considered additional sources for the production of MHC class I peptides increased by inhibition of ubiquitination or proteasomal degradation. Bioinformatic searches identified mitochondrial metalloprotease YME1L1 as enriched in being a putative binding partner of source proteins for the peptides that were increased by proteasome inhibition (fig. S3I). Cleavage by YME1L1 would allow for peptide sampling from mitochondrial inner membrane proteins that are protected from proteasomes. We also found that source proteins for peptides increased by inhibition of ubiquitination were more likely to be reported binders of ubiquitin-binding proteins HRS and GGA1 involved in protein sorting in the trans-Golgi network (fig. S3I) (*22*, *23*). It is possible that some atypical sorting pathway directs these proteins producing MHC class I peptides increased by MLN7243 to lysosomal (or other nonproteasomal) degradation.

It is also possible that proteases in other cellular compartments (e.g., cytosolic, nuclear, and ER) contribute to protein degradation and MHC class I peptide generation in the absence of the UPS. To test this hypothesis, we treated cells with carfilzomib in the presence or absence of an inhibitor of tripeptidyl peptidase II (TPPII) or a combination of inhibitors (E64 and ALLM) targeting multiple proteases, and quantified peptide presentation by mass spectrometry. We observed that treatment with protease inhibitors E64 and ALLM, but not an inhibitor of TPPII, blunted the carfilzomib-induced presentation of select peptides (Fig. 2G and fig. S3J). Therefore, we suspect that multiple intracellular proteases may generate MHC class I peptides in the absence of proteasomal activity, although further studies will be necessary to define the contribution of individual proteases.

### MHC class I peptides only partially dependent on the UPS share unifying characteristics

**Fig. 3.**
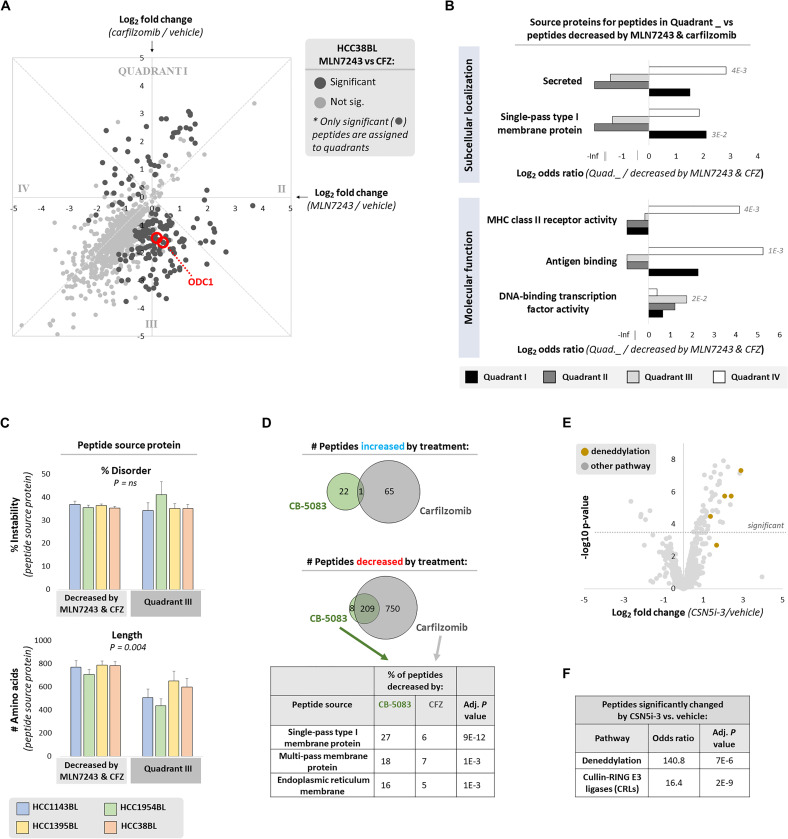
MHC class I peptides only partially dependent on the UPS share unifying characteristics. (**A**) HCC38BL cells were treated for 4 hours with MLN7243 (500 nM; 4-hour pretreatment), carfilzomib (1 μM; 1-hour pretreatment), or vehicle. Peptides not decreased >1.5× by cycloheximide (25 μg/ml; 2-hour pretreatment) were excluded. Dashed perpendicular lines delineate “quadrants”; peptides from the reported proteasome-dependent, ubiquitin-independent substrate ODC1 are marked. (**B**) For each cell line, peptides significantly different between MLN7243 and carfilzomib treatment were assigned to quadrants as in (A). Peptides in each quadrant were compared to peptides significantly decreased by both MLN7243 and carfilzomib. A Cochran-Mantel-Haenszel test was used to test the enrichment of subcellular localization and molecular function terms from UniProt across all cell lines; significant adjusted *P* values are reported. (**C**) In each cell line, proteins assigned to QIII (significantly decreased more by carfilzomib versus MLN7243 treatment) were compared with proteins significantly decreased by both MLN7243 and carfilzomib treatment. The percent disorder and length of each source protein was calculated; significance was determined by Fisher’s method. (**D**) HCC1954BL cells were treated for 4 hours with CB-5083 (5 μM; 1-hour pretreatment), carfilzomib (1 μM; 1-hour pretreatment), or vehicle. Peptides not decreased >1.5× by cycloheximide (25 μg/ml; 2-hour pretreatment) were excluded. Venn diagrams depict the overlap of MHC class I peptides significantly increased or decreased by CB-5083 and carfilzomib treatments. Significance of subcellular localization term enrichment (peptides decreased by CB-5083 versus peptides decreased by carfilzomib) was determined by Fisher’s exact test. (**E**) HCC1954BL cells were treated for 4 hours with MLN4924 (250 nM; 2-hour pretreatment), CSN5i-3 (1 μM; 2-hour pretreatment), or vehicle. Peptides not decreased >1.5× by cycloheximide (25 μg/ml; 2-hour pretreatment) were excluded. (**F**) Enrichment of deneddylation and Cullin-RING E3 ligase (CRL) terms from UniProt in peptides significantly changed by CSN5i-3 versus those not significantly changed was determined by Fisher’s exact test.

Given the differences in localization for MHC class I peptides increased by inhibition of ubiquitination versus proteasomal degradation, we aimed to further define groups of peptides only partially dependent on the UPS for their generation. We identified peptides that differed significantly in their response to MLN7243 or carfilzomib treatment in all cell lines, and grouped these peptides into “quadrants” (Fig. 3A and fig. S4, A and B). These groupings can be generally understood as MHC class I peptides particularly increased by proteasome inhibition (QI) or ubiquitination inhibition (QII), and peptides less ubiquitin-dependent (QIII) and those less proteasome-dependent (QIV). In support of these classifications, reported ubiquitin-independent proteasome substrate ODC1 (*24*) was observed in the expected QIII (Fig. 3A). We then assessed whether subcellular localization and molecular function of MHC class I peptide source proteins in these four quadrants were significantly different from peptides significantly decreased by both MLN7243 and carfilzomib. We observed that peptides in QI were more likely to come from single-pass type 1 membrane proteins, and peptide source proteins in QIV were more likely to be secreted proteins (Fig. 3B). Peptides in QIII were more likely to be from transcription factors, some of which have been previously reported to be ubiquitin-independent substrates (*25*). Peptides in QIV were more likely to be produced from MHC class II proteins; the ubiquitin-dependent, proteasome-independent degradation of these proteins is not surprising, although the immunological significance of these MHC class I peptides is unclear.

Proteasome-dependent, ubiquitin-independent proteins in QIII warranted additional investigation given their surprising number. Source proteins for MHC class I peptides in QIII did not appear to be more disordered as a whole (Fig. 3C) or more likely to be classified as intrinsically disordered (fig. S4C), which has been reported to target proteins to the proteasome independent of ubiquitination (*26*–*30*). Peculiarly, the most marked feature of these proteins is that they are significantly shorter in length (Fig. 3C). The finding of a large collection of MHC class I peptides more dependent on proteasomal degradation than ubiquitination is suggestive of degradation by 20*S* proteasomes dependent on but not bound to 19*S* regulatory particles (typically containing ubiquitin receptors and deubiquitinating enzymes) (*31*). We considered whether other proteasomal features, such as relative abundances of constitutive proteasome versus immunoproteasome subunits (fig. S4, D and E) and expression of proteasome cap proteins beyond 19*S* subunits (fig. S4F), appeared to be strongly associated with differences we observed between the B lymphoblast cell lines we analyzed or differences across the protein degradation treatments tested. We did not observe obvious correlations, although these may be uncovered by further studies analyzing class I peptide presentation across a much larger number of cell lines.

Finally, we were interested in whether MHC class I peptide generation generally requires the segregase/unfoldase p97/VCP. To test this, we treated cells with carfilzomib or the p97 inhibitor CB-5083 and assessed peptide presentation by mass spectrometry. As a whole, MHC class I peptides appeared more dependent on proteasomal degradation than p97 for their generation (Fig. 3D), with less than one-fourth of proteasome-dependent peptides found to also be p97-dependent. However, p97-dependent peptides were preferentially enriched in being derived from multiple types of membrane proteins, as compared with proteasome-dependent peptides. This suggests that the requirement of p97 in canonical MHC class I antigen generation may be limited mostly to proteins requiring extraction from membranes, including ER membrane proteins as expected (*32*), although cross-presentation of exogenous antigens by specialized cell types may also be p97-dependent (*33*).

While inhibition of ubiquitination or proteasomal degradation produces atypical MHC class I peptides (Fig. 2, D and E), sustained complete inhibition of these pathways across different tissue types is not clinically achievable. We considered whether partial inhibition of these pathways might be possible, for example, by inhibiting only the ubiquitination of certain proteins. Cullin-RING (CRL) E3 ligases are responsible for degradation of ~20% of proteins targeted by the proteasome (*34*) and can be selectively inhibited. We treated cells with the neddylation inhibitor MLN4924 and the COP9 signalosome inhibitor CSN5i-3, both of which alter the activity of CRL E3 ligases (fig. S4G). Neither treatment had a large impact on MHC class I peptide presentation (fig. S4H), although it is possible that CRL substrates may be lower in abundance and thus less likely to be detected as peptides by mass spectrometry. Nevertheless, we did observe that CSN5i-3 treatment quite specifically increased presentation of CRL subunits and deneddylation components themselves as MHC class I peptides (Fig. 3, E and F). This effect of CSN5i-3 toward increasing presentation of peptides from these pathways, which is not unexpected given that CSN5i-3 promotes turnover of select CRL complexes (*35*, *36*), speaks to the sensitivity and specificity of our methods and might have clinical applications in cancer patients with known mutations in these genes.

### Atypical MHC class I peptide presentation can be elicited by partial proteasome inhibition

**Fig. 4.**
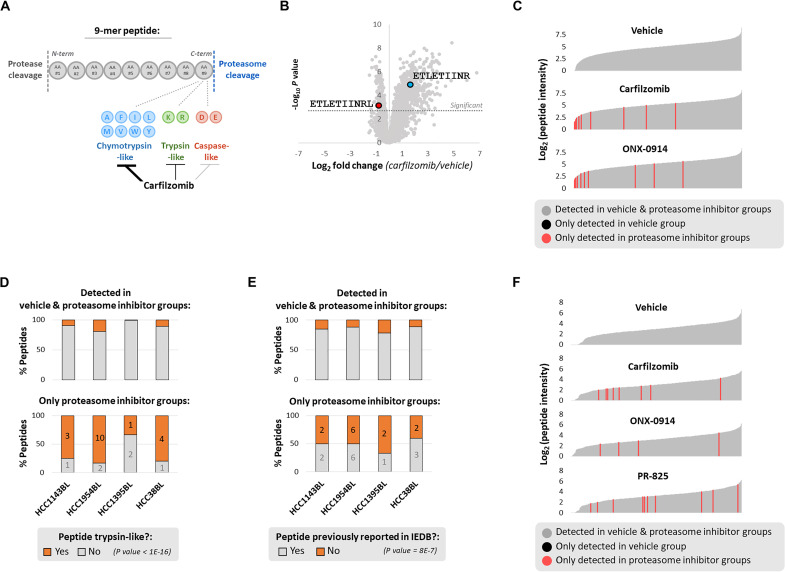
Atypical MHC class I peptide presentation can be elicited by partial proteasome inhibition. (**A**) Predicted impact of proteasome active site usage on MHC class I peptide generation. (**B**) HCC1954BL cells were treated with vehicle or carfilzomib (5 nM) for 48 hours to partially inhibit the proteasome, preferentially its chymotrypsin-like activity. A shift from chymotrypsin activity–like to trypsin activity–like peptide production is depicted. (**C**) HCC1954BL cells were treated for 48 hours with vehicle, carfilzomib (5 nM), or ONX-0914 (25 nM). Waterfall plots depict the log_2_ intensity of nonnormalized MHC class I peptides; peptides are shown as gray lines arranged in increasing intensities. Peptides not detected in vehicle-treated cells (all replicates) but detected in proteasome inhibitor–treated cells (all replicates) are in red. (**D**) Cells were treated as in (C). Plots show MHC class I peptides detected in both vehicle- and proteasome inhibitor–treated cells (all replicates; top), and peptides detected only in proteasome inhibitor–treated cells (all replicates of carfilzomib and ONX-0914 treatment; bottom). A Cochran-Mantel-Haenszel test determined whether peptides only detected in proteasome inhibitor–treated cells were more likely to be trypsin activity–like. Numbers on bottom graph represent number of peptides. (**E**) Similar to (D), peptides bound to MHC class I (HLA-A,-B) previously reported in the Immune Epitope Database (IEDB) are shown in gray; those not previously reported are in orange. A Cochran-Mantel-Haenszel test determined whether peptides only detected in proteasome inhibitor–treated cells were more likely not to have been previously reported in IEDB. (**F**) HCC1954 cells were treated with vehicle, carfilzomib (75 nM), ONX-0914 (200 nM), or PR-825 (250 nM) for 48 hours. Waterfall plots, like those in (C), depict the log_2_ intensity of nonnormalized MHC class I peptides. Peptides only detected in proteasome inhibitor–treated cells (all replicates) are in red.

A method to elicit immunogenic MHC class I peptide display in any patient, agnostic of mutation status, could have substantial clinical implications. We reasoned that this might be achievable via partial inhibition of the proteasome, if atypical peptides can be induced before dose-limiting toxicity is observed. Partial proteasome inhibition could also result in the production of atypical peptides from proteins previously generating MHC class I peptides but now with alternative sequence constraints, due to decreased activity of select proteasome active sites (Fig. 4A).

We were particularly interested in whether atypical MHC class I peptides could be generated under conditions that mimic exposure to proteasome inhibitors in cancer therapy. For these experiments, we chose a dose of carfilzomib that inhibited the chymotrypsin-like site of the proteasome by approximately 75% (fig. S5A). This is comparable to the ≥75% inhibition of the chymotrypsin-like site observed in peripheral blood mononuclear cells of patients 1 hour following carfilzomib treatment (*37*). Because activity of the remaining proteasome active sites (trypsin-like and caspase-like) should remain largely intact under these conditions, we reasoned that overall protein degradation would be only modestly impaired. As a consequence, we anticipated that partial inhibition of the proteasome at the chymotrypsin-like site using clinically relevant concentrations of carfilzomib would alter the repertoire of peptides displayed by MHC class I (Fig. 4A).

We treated B lymphoblast cell lines for 48 hours with carfilzomib, or the immunoproteasome-specific chymotrypsin-like proteasome site inhibitor ONX-0914 (*38*), as immunoproteasomes are dominant in this cell line (fig. S4D). We did not observe compensatory up-regulation of other proteasome active sites in response to these treatments (fig. S5, B and C) or changes in proteasome subunit expression except for decreased expression of alternative proteasome cap PA200 (fig. S5D). We classified MHC class I peptides identified by mass spectrometry according to which proteasomal active site, if any, we predicted they would be produced by (fig. S5E).

Whereas complete proteasome inhibition greatly reduced MHC class I peptide display (Fig. 2A and fig. S2H), we observed that, with the exception of a small fraction of peptides that were significantly decreased (fig. S5G), peptide display was generally unchanged or mildly up-regulated by partial proteasome inhibition (fig. S5F). As predicted, MHC class I peptides significantly increased by partial proteasome inhibition targeting the chymotrypsin-like site, as compared to those decreased, had C termini more likely to be produced by trypsin-like proteasomal cleavage (fig. S5H). The only exception to this was in HCC1395BL cells, which typically do not present trypsin activity–like peptides due to MHC class I allele binding preferences (fig. S5E). This shifting from proteasomal chymotrypsin-like site activity to trypsin-like site activity could occasionally be observed for overlapping peptides (Fig. 4B); we expect that this would be observed more frequently in mass spectrometry experiments with deeper coverage.

While non–chymotrypsin activity–like peptides were more likely to be significantly increased by proteasome inhibition, most of these peptides were also detected in vehicle-treated cells, and thus seem unlikely to be immunogenic. However, we were able to identify a small number of peptides only detected upon partial proteasome inhibition (Fig. 4C). As expected, these peptides were more likely to be produced by the non–chymotrypsin-like sites of the proteasome (Fig. 4D). They were also more likely not to have been previously reported as MHC class I antigens in the Immune Epitope Database (*39*) (Fig. 4E), hence increasing their potential for immunogenicity.

The idea that MHC class I peptides produced by mild proteasome inhibition, like that observed clinically, might be immunogenic is difficult to reconcile with the fact that patients treated with these inhibitors do not appear to exhibit self-directed immune responses. However, proteasome inhibitors used clinically may also suppress the function of immune cells required for a response to atypical peptides. We considered that one way to bypass this limitation would be to treat solid tumor cells, which are constitutive proteasome dominant, with a proteasome inhibitor that specifically inhibits only the constitutive proteasome and thus would have little effect on immune cells that contain high levels of immunoproteasome.

To test this idea, we used HCC1954 breast cancer cells derived from the same patient as the HCC1954BL B lymphoblast cell line (*40*). Unlike HCC1954BL, HCC1954 cells expressed approximately twice as many constitutive proteasome subunits as immunoproteasome subunits (fig. S6, A and B). We aimed to treat these cells with carfilzomib, which targets constitutive and immunoproteasomes, as well as the immunoproteasome-specific inhibitor ONX-0914 and constitutive proteasome-specific inhibitor PR-825 (*41*) at doses that selectively inhibited the chymotrypsin-like site of the proteasome/immunoproteasome. As it is difficult to distinguish immunoproteasome versus constitutive proteasome inhibition in vivo, we assumed complete specificity of ONX-0914 and PR-825 and estimated the percent of chymotrypsin-like proteasome activity inhibition to be indicative of immunoproteasome/constitutive proteasome (fig. S6B). Complete inhibition of the chymotrypsin-like site with carfilzomib was too toxic to obtain; however, we expect to have inhibited the chymotrypsin-like activity of the immunoproteasome or constitutive proteasome completely with ONX-0914 or PR-825, respectively, with minimal off-target activity (fig. S6C). HCC1954 cells were treated with these doses of carfilzomib, ONX-0914, and PR-825 for 48 hours and MHC class I peptide presentation assessed by mass spectrometry. As in HCC1954BL cells, overall peptide presentation was not reduced by these inhibitors (fig. S6D); instead, presentation of select peptides was enhanced (fig. S6, E and F), particularly with carfilzomib and PR-825 treatment. Also, as observed in HCC1954BL cells, certain peptides were only detected in proteasome inhibitor–treated cells (Fig. 4F). That these peptides were observed in PR-825–treated cells further suggests that partial proteasome inhibition using constitutive proteasome-specific inhibitors may enhance immunogenic MHC class I peptide presentation in solid tumor cells while minimizing potential negative impacts on effector immune cells.

## DISCUSSION

Although the UPS is widely thought to play a role in MHC class I presentation, the nature and extent of its role has provoked some controversy. The strongest evidence for a role for the UPS comes from studies that quantify total levels of cell surface MHC class I following chemical inhibition of the UPS, supplemented by analysis of presentation of select model MHC class I–binding peptides in response to UPS inhibition (*3*–*5*). While collectively these analyses indicate a role for the UPS in peptide presentation, they do not reveal the contribution of specific pathways within the UPS, nor do they provide insight into the identities of the peptides that are dependent or independent of those pathways. Other groups have sought to address these points by using mass spectrometry to take a census of MHC class I–bound peptides under different conditions. This has spawned unresolved ambiguity, particularly related to the role of the proteasome in MHC class I peptide presentation. The first group investigating this question arrived at the surprising and unrefuted claim that there is little or no role for the proteasome in presentation of most MHC class I peptides (*7*). Conversely, a later report from a second group supported a major role for the proteasome (*42*), although they did not rationalize their results with those of Milner *et al.* and their claims were limited by the semiquantitative nature of their mass spectrometry experiments.

To address this discrepancy, we first sought to address technical limitations of previous studies that quantified MHC class I peptide presentation by mass spectrometry by developing methodology to enhance the quality of our data (Fig. 1), including the use of a spike-in MHC class I peptide standard to enable more accurate normalization and a computational approach to exclude background peptides that are generally of low abundance and unresponsive to inhibitors that broadly block MHC class I peptide presentation. Our approach enabled us to produce the most extensive catalog of UPS-dependent MHC class I peptides generated to date. From our work across multiple cell lines, we concluded that most MHC class I•peptide complexes require the proteasome (~70%) and ubiquitination (~60%) for their generation (Fig. 2). An explanation for the discordant results regarding the role of the proteasome in MHC class I peptide generation observed here versus previously reported (*7*) is that the previous work prioritized minimizing proteasome inhibitor toxicity at the expense of achieving complete proteasome inhibition. What we termed “partial proteasome inhibition,” for which we did not observe overall decreased MHC class I peptide presentation (Fig. 4), better reflects the extent of proteasome inhibition this group achieved.

One unexpected finding of our studies was that of the ~70% of class I–bound peptides that were dependent on proteasome activity, close to 20% were formed independently of ubiquitin conjugation activity (Fig. 3). These peptides were enriched in being from transcription factors, which previous studies have suggested can serve as a source of ubiquitin-independent substrates (*25*). Ubiquitination-independent peptides did not appear more likely to be from disordered proteins, but instead were derived from significantly shorter source proteins. The significance of this latter observation is unclear. It will be interesting to see if ubiquitin-independent peptides are generated by 26*S* proteasomes [as is the case for ubiquitin-independent ODC1 (*24*)], free 20*S* proteasomes, which degrade nonubiquitinated hydrophobic peptides (*43*) and oxidized or disordered proteins in vitro (*30*, *44*) and in vivo (*45*), or 20*S* proteasomes bearing alternative caps (e.g., PA28αβ, PA28γ, and PA200) (*46*).

Another component of the UPS that contributes to generation of a subset of class I–bound peptides is the segregase/unfoldase p97/VCP. We found that p97 is required for the generation of ~20% of presented peptides that are proteasome-dependent, particularly those from membrane proteins (Fig. 3). This might be expected from the known roles of p97 in ER-associated degradation and membrane protein extraction (*47*), although p97 may also be involved in cross-presentation of antigens from the endolysosomal system in specialized cell types. In contrast to p97, we observed little involvement of Cullin-RING ubiquitin ligase (CRL) activity in generation of MHC class I•peptide complexes, despite these enzymes being involved in up to 20% of proteasome-dependent protein degradation (*34*). Perhaps CRL substrates are, on average, too inabundant to be detected at the level of coverage achieved in our mass spectrometry experiments. Of note, CSN5i-3, a drug that binds and inhibits COP9 signalosome (CSN) and thereby stimulates turnover of a subset of CRL subunits (*35*), led to increased presentation of CSN and CRL subunits. Given that CRL subunits are relatively inabundant, this remarkable observation underscores the sensitivity and specificity of our methodology and its ability to detect more subtle perturbations to the UPS.

In addition to the prominent role for the UPS, a significant finding that emerged from our work was evidence for UPS-independent formation of MHC class I•peptide complexes. We observed constitutive presentation of UPS-independent peptides disproportionately derived from mitochondrial inner membrane proteins, the mature forms of which are normally inaccessible to the UPS, although the mechanisms of their generation remain unclear. Another finding is that a smaller number of peptides (~5%) are paradoxically increased by inhibition of UPS pathways. These peptides tend to be preferentially derived from mitochondrial proteins upon UPS inhibition and single-pass transmembrane proteins upon proteasome inhibition alone. Although compensatory autophagy can occur in response to decreased proteasomal activity (*48*), autophagy did not appear to be a major source of class I–displayed peptides increased by proteasome inhibition, as induction of these peptides was not reduced by the lysosomal protease inhibitor leupeptin or an ATG7 inhibitor.

We then wondered whether increased presentation of atypical MHC class I peptides upon proteasome inhibitor treatment could also occur in cancer therapeutic contexts where only partial proteasome inhibition is achievable (*49*, *50*). To address this, we examined peptide presentation under conditions achieved upon carfilzomib treatment in blood cancer patients, in which the chymotrypsin-like site of the proteasome is inhibited by ~75%, with much lesser inhibition of the trypsin-like and caspase-like sites (*37*). Under these conditions, at least some protein degradation is expected to occur, but there should be a shift in the spectrum of peptides produced. Partial proteasome inhibition did not decrease overall peptide presentation; in fact, certain peptides were increased in presentation by partial proteasome inhibition, particularly those produced by noninhibited proteasome active sites (Fig. 4). We were especially interested in peptides detected only in proteasome inhibitor–treated cells, which were rare (generally <10 detected per cell line) but significantly more likely not to have been previously reported. Additionally, we confirmed in a breast cancer cell line that proteasome inhibitor–specific peptides could be induced by proteasome inhibitors currently in the clinic (carfilzomib), as well as inhibitors selective for the immunoproteasome and constitutive proteasome (ONX-0914 and PR-825, respectively). While selective inhibitors of the constitutive proteasome have not been developed for clinical usage, our results suggest that these could have application in cancer immunotherapy, since alternative and perhaps immunogenic peptide display could potentially be induced in solid tumor cells without affecting the viability or function of effector immune cells, which primarily express immunoproteasomes. Off-target presentation of immunogenic MHC class I peptides by nontumor cells could limit the therapeutic tolerability of this approach, although off-target toxicity can also be observed following checkpoint inhibitor treatment (*51*).

Notably, increased presentation of atypical MHC class I peptides was also observed upon inhibition of additional UPS pathways. It is possible that a larger number of unconventional compensatory protein degradation pathways, in the absence of canonical degradation pathways, could similarly generate potentially immunogenic MHC class I peptides. For example, targeting a proteasomal substrate for endolysosomal degradation (*52*) or a 26*S* proteasomal substrate for 20*S* proteasomal degradation (*45*) is expected to alter the sequence of protein degradation fragments generated. It has also been reported that immunoproteasomes, which are inducible, generate MHC class I peptides distinct from those generated by the constitutive proteasome (*41*, *53*), although this is controversial (*54*).

**Fig. 5.**
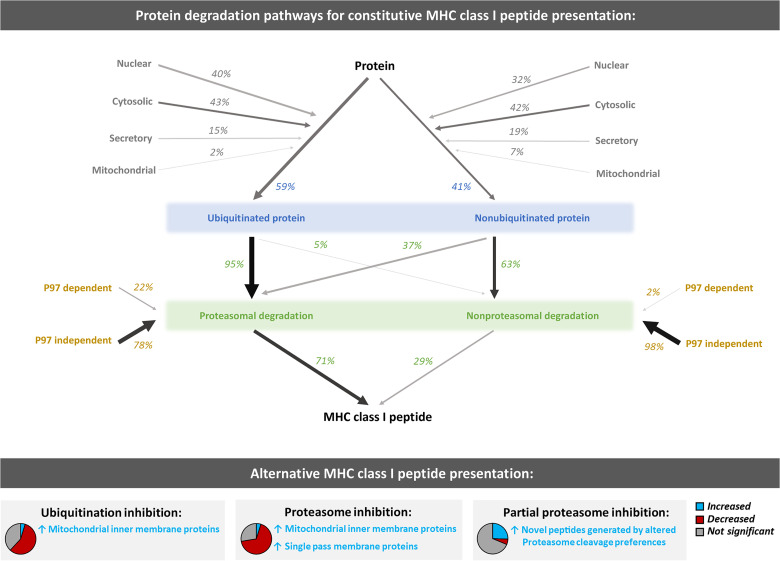
Diverse constitutive and alternative protein degradation pathways produce MHC class I peptides. Summary of impact of protein degradation pathway inhibition on MHC class I peptide generation. In the top panel, a quantitative summary of constitutive protein degradation pathways generating MHC class I peptides is depicted. Numbers represent percent of peptides found to be significantly decreased in presentation by inhibition of indicated protein degradation pathways (original data in Figs. 2A and 3D). In the bottom panel, pie charts represent relative numbers of increased (blue), decreased (red), and not significantly changed (gray) peptides. Notable characteristics of significantly increased peptides (blue) are provided as text.

In summary, we provide the most comprehensive quantitative analysis of the source of peptides loaded onto MHC class I molecules (Fig. 5). This work serves as a baseline resource for understanding the myriad mechanisms underlying the generation of the MHC class I repertoire, which is fundamental to the ability of our adaptive immune systems to distinguish self from nonself. An interesting goal for future work will be to apply methods like those reported here to determine in greater mechanistic detail how individual pathways in the UPS contribute to the class I peptide repertoire and to understand how genetic or environmental factors associated with the development of human inflammatory, infectious, or neoplastic diseases influence this repertoire.

## METHODS

### Study design

This study aimed to determine the contribution of diverse protein degradation pathways to generation of MHC class I peptides using quantitative mass spectrometry. Multiplexed B lymphoblast samples treated with chemical inhibitors were generated for the following experiments: inhibition of MHC class I secretion (HCC1954BL cell line; this cell line was used for all other experiments unless otherwise noted); inhibition of translation, MHC class I secretion, and removal of cell surface MHC class I by acidic peptide elution (HCC1954BL and HCC38BL cell lines); inhibition of ubiquitination and the proteasome (HCC1143BL, HCC1954BL, HCC1395BL, and HCC38BL cell lines); DNA damage and apoptosis induction; inhibition of autophagy in combination with proteasome inhibition (two separate experiments using leupeptin or an ATG7 inhibitor); inhibition of the proteasome in combination with inhibition of TPPII or protease inhibitors; inhibition of disaggregase p97/VCP; modulation of Cullin-RING E3 ligase activity; and partial inhibition of the proteasome with carfilzomib and an immunoproteasome inhibitor (HCC1143BL, HCC1954BL, HCC1395BL, and HCC38BL cell lines). Multiplexed HCC1954 breast cancer cell line samples were also generated that were treated with carfilzomib, an immunoproteasome inhibitor, and a constitutive proteasome inhibitor for partial proteasome inhibition.

### Cell lines

HCC1143BL, HCC1954BL, HCC1395BL, HCC38BL, and HCC1954 (*40*) were purchased from the American Type Culture Collection and cultured in RPMI 1640 containing penicillin-streptomycin and 10% fetal bovine serum (FBS). For experiments, B lymphoblast cell lines were used at a concentration of approximately 1 × 10^6^ cells/ml.

For certain experiments using B lymphoblasts, cells were pretreated with protein degradation inhibitors or vehicle for times indicated, and cell surface MHC class I was removed by acid stripping. This was accomplished by bathing approximately 2 × 10^7^ cells in 10 ml of acid stripping buffer (0.132 M citric acid, 0.06 M sodium phosphate, pH 3.0) for 2 min on ice, followed by neutralization in 40 ml of ice-cold medium. Cells were washed once in medium before experimental treatment.

Cells were collected for mass spectrometry by centrifugation, which was preceded by trypsin detachment for adherent cell lines (HCC1954). Cell pellets were washed once in phosphate-buffered saline (PBS), and cell count and viability were assessed using Vi-CELL XR.

### Inhibitors

Carfilzomib, MLN4924, and CB-5083 were purchased from Selleck Chemicals. MLN7243 was purchased from Chemie Tek. Cycloheximide, E64, and ALLM were purchased from Sigma-Aldrich. AAF-CMK was purchased from Enzo Life Sciences. Brefeldin A and leupeptin were purchased from Alfa Aesar. Cisplatin was purchased from R&D Systems. CSN5i-3 and ONX-0914 were purchased from Thermo Fisher Scientific. PR-825 and the ATG7 inhibitor, compound #37 (*21*), were synthesized by WuXi AppTec.

### Spike-in SIINFEKL standard

Mouse DC2.4 dendritic cells were purchased from Millipore and maintained in RPMI 1640 containing 10% FBS, penicillin-streptomycin, and l-glutamine. Purified SIINFEKL peptide from ovalbumin protein (AnaSpec) was added to cells at 25 μg/ml for 4 hours. Cells were washed in PBS and stored as frozen pellets for future mass spectrometry experiments.

### Flow cytometry quantification of cell surface MHC class I

Cells were washed twice in PBS. Approximately 1 × 10^5^ cells were resuspended in 100 μl of 2% FBS in PBS with anti–MHC class I antibody (W6/32-PE) at a 1:50 dilution. Cells were incubated at 4°C for 30 min and then washed three times in 2% FBS in PBS. Flow cytometry was performed on BD Symphony (MHC class I expression following acid stripping) or BD LSR II (MHC class I expression following MLN7243, carfilzomib, and cycloheximide treatment in HCC1143BL and HCC1954BL cells). Experiments were gated on single cells, and 10,000 events were captured per sample. Median fluorescence intensity of phycoerythrin (PE) was calculated by BD FACSDiva.

### Proteasome activity assay

Approximately 5 × 10^5^ cells were washed in PBS and lysed in 500 μl of cold assay buffer [50 mM Hepes (pH 7.8), 10 mM NaCl, 1.5 mM MgCl_2_, 1 mM EDTA, 1 mM EGTA, 250 mM sucrose, 5 mM dithiothreitol]. Cells were vortexed and sonicated for 5 s at 50% power and then centrifuged at 14,000 rpm for 10 min at 4°C. The lysate (50 μl) was loaded onto a 96-well plate on ice in triplicate for each proteasome activity probe. Assay buffer (200 μl) containing 2 mM adenosine triphosphate (ATP) and 100 μM substrate (Suc-LLVY-AMC, Boc-LRR-AMC, Z-LLE-AMC, or Ac-nLPnLD-AMC) in dimethyl sulfoxide (DMSO) was added to each well, and plates were incubated at 37°C for 1 hour. Plates were then imaged on a SpectraMax M5 fluorescent plate reader (excitation: 355 or 360 nM; emission: 460 nM). Fluorescence intensity was normalized to total protein content calculated by Bradford assay or total cell number.

### Quantification of translation

HCC1954BL cells were treated with 500 nM MLN7243 (4 hours), 1 μM carfilzomib (1 hour), cycloheximide (25 μg/ml) (2 hours), or vehicle. For the final 30 min of treatment, cells were treated in methionine-deficient RPMI 1640 with 10% dialyzed FBS and penicillin-streptomycin. Cells (1 × 10^6^) were then labeled with [^35^S]methionine (25 μCi/ml) (PerkinElmer NEG709A001MC) for 5 min. Unlabeled l-methionine (1 mg/ml) was added as previously described (*55*) to terminate radiolabeling, and cells were centrifuged at 300*g* for 1 min. Cells were washed with cold PBS containing l-methionine (1 mg/ml) twice. Cells were then lysed in cold radioimmunoprecipitation assay (RIPA) buffer for 5 min on ice and centrifuged at 14,000 rpm for 5 min. Proteins from supernatants were precipitated by adding 20% cold trichloroacetic acid on ice for 1 hour, followed by centrifugation at 14,000 rpm for 5 min. Pellets were washed with cold 5% trichloroacetic acid and subsequently cold acetone, with pelleting by centrifugation at 14,000 rpm for 5 min. Pellets were then resuspended in RIPA buffer and mixed with scintillation fluid. Incorporation of radiolabeled methionine was measured using a Beckman Coulter LS6500 scintillation counter set to read [^35^S] for 1 min.

### Purification of MHC class I peptides

MHC class I peptides were purified from frozen samples based on a protocol described previously (*13*). Before purification, anti-human MHC class I antibody (W6/32; Bio X Cell) was crosslinked to protein A–Sepharose 4B beads by incubation for 1 hour at room temperature with shaking, followed by crosslinking in 20 mM dimethyl pimelimidate dihydrochloride and 0.1 M sodium borate for 30 min at room temperature. Equal parts 0.2 M ethanolamine (pH 8) was added and mixed for 5 min. Solution was removed from beads, and beads were incubated with 0.2 M ethanolamine (pH 8) for 2 hours at room temperature. Beads were washed three times in PBS and stored in an equivalent volume of PBS with 0.02% sodium azide. Similarly, antibody against mouse MHC class I H-2K^b^ bound to SIINFEKL peptide (25-D1.16; Bio X Cell) was separately crosslinked to protein A–Sepharose 4B beads. Anti-human MHC class I antibody beads were mixed with anti-mouse MHC class I•SIINFEKL beads at a 100:1 ratio.

Approximately 1 × 10^7^ frozen human cells were lysed in 1 ml of cold lysis buffer [PBS with 0.25% sodium deoxycholate, 0.2 mM iodoacetamide, 1 mM EDTA, protease inhibitor cocktail, 1 mM phenylmethylsulfonyl fluoride (PMSF), and 1% octyl-β-d-glucopyranoside]. Frozen mouse cells presenting the SIINFEKL spike-in standard were also lysed in this buffer. Cells were lysed on ice with occasional vortexing for 30 min, and then lysates were centrifuged at 14,000 rpm for 30 min at 4°C. During this time, a 96-well filter plate was washed with 200 μl of acetonitrile and 200 μl of 0.1% formic acid, and twice with 200 μl of 0.1 M tris-HCl (pH 8). Plates were centrifuged at 200 rpm for 1 min at 4°C if needed.

For each experiment, cleared lysate volumes representing an identical number of cells were used. These lysates were mixed with mouse cells presenting SIINFEKL peptide at a ratio of 100:1 cells. Antibody slurry (150 μl) was added to wells of the 96-well filter plate and washed with 200 μl of lysis buffer. Lysates were then passed through wells containing antibodies by gravity flow. Wells were washed four times with 200 μl of cold 150 mM NaCl in 20 mM tris-HCl (pH 8), four times with 200 μl of cold 400 mM NaCl in 20 mM tris-HCl (pH 8), four times with 200 μl of cold 150 mM NaCl in 20 mM tris-HCl (pH 8), and two times with 200 μl of cold 20 mM tris-HCl (pH 8). Plates were centrifuged at 200 rpm for 1 min at 4°C to pass wash buffers through plate. During this time, a Waters Sep-Pak tC18 96-well plate was washed with 1 ml of 80% acetonitrile in 0.1% formic acid, followed by 2 ml of 0.1% formic acid. MHC class I complexes were eluted from the antibody plate into the C18 plate with 500 μl of 1% trifluoroacetic acid. The C18 plate was washed with 2 ml of 0.1% formic acid, and MHC class I peptides were eluted with 500 μl of 28% acetonitrile in 0.1% formic acid.

Purified peptides were dried using a GeneVac vacuum evaporator and resuspended in 100 mM Hepes (pH 8). Peptides were N-terminally labeled using TMT labels (10 samples: TMT10plex; 11 samples: TMT10plex + TMT11-131C; 12 to 16 samples: TMTpro) and combined for a single mass spectrometry run. Peptides were dried and desalted using C18 10 μl ZipTips before analysis.

### LC-MS analysis using SPS-MS3

For most experiments, the entire sample was used for a single mass spectrometry run. Labeled peptides were subjected to liquid chromatography–tandem mass spectrometry (LC-MS/MS) analysis on an EASY 1000 nanoflow LC system coupled to a Fusion Tribrid Orbitrap mass spectrometer (Thermo Fisher Scientific) or an Eclipse Tribrid Orbitrap mass spectrometer (Thermo Fisher Scientific) equipped with a Nanospray Flex ion source. Samples were directly loaded onto an Aurora 25 cm × 75 μm inner diameter (ID), 1.6 μm C18 column (Ion Opticks) heated to 50°C. The peptides were separated with a 2-hour gradient at 350 nl/min as follows: 2 to 6% solvent B (7.5 min), 6 to 25% B (82.5 min), 25 to 40% B (30 min), 40 to 98% B (1 min), and held at 98% B (15 min). Solvent A consisted of 97.8% H_2_O, 2% acetonitrile (ACN), and 0.2% formic acid, and solvent B consisted of 19.8% H_2_O, 80% ACN, and 0.2% formic acid. The Fusion was operated in data-dependent mode. Spray voltage was set to 2.2 kV, S-lens radio frequency (RF) level at 60, and heated capillary at 275°C. Full-scan resolution was set to 120,000 at mass/charge ratio (*m*/*z*) 200 in Profile mode with an automatic gain control (AGC) target of 4 × 10^5^ and a maximum injection time of 50 ms. Precursor mass range was set to 400 to 1500 *m*/*z*, and the isolation window was set to 0.7 *m*/*z*. For data-dependent MS2 scans, the cycle time was 3 s, AGC target value was set at 5 × 10^4^, and intensity threshold was kept at 5 × 10^3^. Collision-induced dissociation (CID) fragmentation of precursors was performed with a fixed collision energy of 35%, activation time of 10 ms, and activation Q of 0.25. MS2 scans were then performed in the Orbitrap at 30,000 resolution in Centroid mode using auto scan range and a maximum injection time of 150 ms. Dynamic exclusion was enabled to exclude after two times for 60 s with a mass tolerance of 10 parts per million (ppm). A charge state filter was also applied to only include precursors of charge 2 to 5. Quantitative MS3 scans were then performed using multi-notch isolation. Synchronous precursor selection (SPS) precursors were selected from the mass range of 400 to 1600 *m*/*z* with a precursor ion exclusion window from −50 to +5 *m*/*z*. Quadrupole isolation of the precursor used an isolation window of 0.7 *m*/*z*, while the ms2 isolation window was set to 3 *m*/*z* for up to 10 notches. The AGC target was 5 × 10^4^, and the maximum injection time was set to 500 ms. Higher-energy collisional dissociation (HCD) fragmentation was performed with fixed collision energy of 65% followed by Orbitrap detection at 50,000 resolution in Centroid mode using a scan range from 100 to 500 *m*/*z*.

### Mass spectrometry data search

Raw data were analyzed in Proteome Discoverer 2.4 (Thermo Fisher Scientific) using an unspecific (no-enzyme) search with the Byonic search algorithm (Protein Metrics) and UniProt human fasta file containing the spike-in peptide sequence SIINFEKL. PD-Byonic search parameters were as follows: precursor mass tolerance of 5 ppm, CID low-energy fragmentation, fragment mass tolerance of 20 ppm, and a maximum of two common modifications and one rare modification. Cysteine carbamidomethylation and TMT-6 or TMTpro addition to peptide N termini and lysine were set as static modifications. Methionine oxidation was a common dynamic modification (up to two per peptide), and deamidated asparagine or glutamine was set as a rare dynamic modification (only one per peptide). Precursor and charge assignments were computed from MS1. Byonic protein-level false discovery rate (FDR) was set at 0.01, while Percolator FDRs were set at 0.001 (strict) and 0.01 (relaxed). In the consensus workflow, peptide and peptide-spectrum match (PSM) FDRs were also set at 0.001 (strict) and 0.01 (relaxed), with peptide confidence at least medium, lower confidence peptides excluded, minimum peptide length set at 7, remove peptides without a protein reference set to false, and apply strict parsimony set to true. Quantification was performed at the ms3 level using reporter ion signal-to-noise (S/N) ratios with an average reporter S/N threshold of 35, a co-isolation threshold of 30%, and an SPS mass matches threshold of 70%.

### Mass spectrometry data statistical analysis

PSM output files from ProteomeDiscoverer were filtered for peptides not flagged in “Quan Info” as “ExcludedByMethod.” Only peptides 7 to 14 amino acids long were used. Peptides also needed to have an abundance quantitation for at least three replicates of one treatment to be used. After this filtering, missing values were rare; when they occurred, values were imputed from a Gaussian distribution centered on the bottom 1st peptide intensity percentile with SD of the median SD of peptides in the bottom 10th intensity percentile. PSMs for identical sequence peptides from the same UniProt ID, disregarding location of TMT labeling and modification, were averaged. Peptide intensities were then normalized to the spike-in standard SIINFEKL intensity.

A limma test was performed for statistical significance between treatments with R (*56*). Results were considered significant when the adjusted *P* value was <0.01. After performing the limma test, negative values from Gaussian imputation were changed to the dataset minimum value so that log_2_ fold changes could be calculated.

In certain experiments, an additional filter was applied: only peptides decreased at least 1.5 fold by cycloheximide treatment in both replicates. This filter was applied after the limma test was performed. Heatmaps were generated using Seaborn’s clustermap function with no column clustering in Python.

### Enrichment of subcellular localization and molecular function terms

The number of proteins associated with GO Cellular Compartment and GO Molecular Function terms was obtained from GeneTrail2 (*57*). Subcellular localization terms were also obtained from UniProt (*58*). Simplified subcellular localization terms, excluding “unknown,” were obtained from SubCellBarCode (*59*). Enrichment of terms for MHC class I peptide nonredundant source proteins from one group of interest was compared with a second (e.g., nonredundant source proteins for peptides significantly increased by carfilzomib versus those significantly decreased). For experiments performed in a single cell line, enrichment of these terms was determined by Fisher’s exact test with Bonferroni multiple testing corrections. At least five proteins needed to be associated with the term in at least one of the groups of peptides to be considered. For experiments performed in multiple cell lines, enrichment of these terms across cell lines was determined by a Cochran-Mantel-Haenszel test with Bonferroni multiple testing corrections. At least five proteins across all cell lines needed to be associated with the term in at least one of the groups of peptides to be considered.

### Enrichment of protein-protein interactions

Proteins reported to bind MHC class I peptide source proteins were obtained from BioGRID (*60*). Enrichment of interactors for peptide nonredundant source proteins from one group of interest was compared with a second (e.g., nonredundant source proteins for peptides significantly increased by carfilzomib versus those significantly decreased). Only low-throughput, physical interactors were used. Enrichment of these interactors across cell lines was determined by a Cochran-Mantel-Haenszel test with Bonferroni multiple testing corrections. At least five proteins across all cell lines needed to be associated with the interactor in at least one of the groups of peptides to be considered.

### Assessment of protein degradation by cycloheximide chase

HCC1954BL cells were pretreated with vehicle or carfilzomib (1 μM) for 10 min. Cycloheximide (25 μg/ml) was then added, and cells were collected at 0-, 1-, and 4-hour time points. Known UPS substrates were obtained from UbiNet 2.0 (*61*). Of these proteins, the five proteins with the shortest reported half-lives in B cells (*62*) were chosen for immunoblotting: JAK3, AMFR, JAK1, SQSTM1, ETV5, and IRF8. SQSTM1 was removed from consideration since autophagy can be induced by proteasome inhibition (*48*). JAK3 did not decrease in response to cycloheximide in vehicle-treated cells by the 4-hour time point and thus was not included.

### Immunoblotting

Cells were lysed in NP-40 lysis buffer [50 mM tris-HCl (pH 7.4), 150 mM NaCl, 1% NP-40] for the following experiments: treatment of cells with leupeptin or the ATG7 inhibitor, treatment of cells with MLN4924 or CSN5i-3, and treatment of cells with vehicle or carfilzomib in combination with cycloheximide. Cells were lysed in lysis buffer used for MHC class I peptide mass spectrometry (PBS with 0.25% sodium deoxycholate, 0.2 mM iodoacetamide, 1 mM EDTA, protease inhibitor cocktail, 1 mM PMSF, and 1% octyl-β-d-glucopyranoside) for the following experiment: dose and time response treatment with MLN7243. Protein concentration was determined by bicinchoninic acid (BCA) assay (MHC class I peptide mass spectrometry lysis buffer) or Bradford assay (NP-40 lysis buffer). Equivalent protein amounts were run on tris-glycine gels and transferred to nitrocellulose membranes using Teknova Electroblot Buffer. Membranes were blocked in 5% milk, and primary antibodies were used at 1:1000 concentration.

Antibodies used were to polyubiquitin (FK1; Enzo BML-PW8805-0500), K48 polyubiquitin (Millipore 05-1307), ubiquitinated H2B (Cell Signaling 5546S), β-actin (Cell Signaling 3700S), LC3B (Cell Signaling 2775S), p62 (Cell Signaling 5114S), CUL1 (Invitrogen 71-8700), AMFR (Cell Signaling 9590S), JAK1 (Cell Signaling 29261S), ETV5 (Cell Signaling 16274S), IRF8 (Cell Signaling 83413T), PSMB5/β5 (Cell Signaling 12919S), PSMB8/LMP7 (Cell Signaling 13635S), RPN11/PSMD14 (Sigma HPA002114), PSME1/PA28α (Novus NBP1-83121), PA28β (Cell Signaling 2409S), PA28γ (Cell Signaling 2412S), and PSME4/PA200 (Novus NBP2-22236).

### Flow cytometry measurement of apoptosis

HCC1954BL cells were treated with vehicle, 50 μM cisplatin, or 500 μM cisplatin for 20 hours, acid-stripped to remove preexisting MHC class I complexes, and treated again with vehicle, 1 μM carfilzomib, 50 μM cisplatin, or 500 μM cisplatin for 4 hours. Approximately 1 × 10^6^ cells were stained with annexin V–FITC (fluorescein isothiocyanate) and propidium iodide (PI), staining apoptotic and dead cells, respectively, using a Life Technologies Dead Cell Apoptosis kit. Cutoffs for annexin V–FITC and PI staining were established using unstained cells. Cells were classified as “live” (below the cutoff for annexin V–FITC and PI staining), “early apoptotic” (above the cutoff for annexin V–FITC staining), “late apoptotic/necrotic” (above the cutoff for annexin V–FITC and PI staining), and “other.” Experiments were gated on single cells, and 10,000 events were captured per sample on BD Symphony. Analysis was performed with FACSDiva.

### Fluorescent proteasome gels

One hour before cell harvesting, cells were treated with 500 nM Me4BodipyFL-Ahx3Leu3VS. Cells were washed with PBS during harvesting and lysed in cold NP-40 lysis buffer [50 mM tris-HCl (pH 7.4), 150 mM NaCl, 1% NP-40, and protease inhibitor cocktail]. Cells were lysed at 4°C for 30 min with rotation and centrifuged at 14,000 rpm for 5 min at 4°C. Protein (10 μg) was loaded onto 16% tricine SDS-PAGE gels and run for approximately 4 to 6 hours at 120 V with tricine running buffer containing 1:400 NuPAGE antioxidant. Gels were imaged on a Typhoon FLA9500 fluorescent gel scanner (excitation: 473 nm; emission filter: BPB1 530DF20).

### Enrichment of deneddylation and Cullin-RING ligase terms after CSN5i-3 treatment

Proteins with deneddylation gene ontology terms were obtained from UniProt in November 2020. Proteins listed as Cullin-RING ligases (CRL) were obtained from UUCD (*63*). Nonredundant source proteins for peptides significantly increased by CSN5i-3 treatment were compared with nonredundant source proteins for peptides not significantly changed by CSN5i-3 treatment. Enrichment of these terms in peptides significantly increased by CSN5i-3 treatment was determined by Fisher’s exact test with Bonferroni multiple testing corrections.

### Proteasome cleavage site prediction

The C-terminal amino acid was determined for MHC class I peptides and classified as “chymotrypsin activity–like” (alanine, phenylalanine, isoleucine, leucine, methionine, valine, tryptophan, or tyrosine), “trypsin activity–like” (lysine or arginine), or “other” (all other amino acids) (*64*). Enrichment of trypsin activity–like peptides in peptides increased by ONX-0914 versus those decreased was determined by Fisher’s exact test. Enrichment of trypsin activity–like peptides in peptides only detected after proteasome inhibitor treatment across all cell lines was determined by a Cochran-Mantel-Haenszel test.

### Calculation of MHC class I peptide source protein length and disorder

MHC class I peptide source protein length was obtained from UniProt. The percent instability of peptide source proteins was obtained from Dryad (*65*, *66*). Significance of differences in length and percent disorder between nonredundant proteins in QIII and those decreased by MLN7243 and carfilzomib treatment across all cell lines was determined by Fisher’s method combining *t* test *P* values from each cell line. The number of peptide source proteins considered to be intrinsically disordered was obtained from DisProt (*67*). Significance of differences in fraction of nonredundant peptide source proteins characterized as intrinsically disordered in QIII versus those decreased by MLN7243 and carfilzomib treatment (UPS-dependent) across all cell lines was determined by a Cochran-Mantel-Haenszel test.

### Calculation of differences in protein abundance and half-life between UPS-dependent and UPS-independent MHC class I peptide source proteins

For UPS-dependent and UPS-independent MHC class I peptide source proteins, the reported half-lives in B cells (*62*) and reported protein abundance in B cells (*68*) were determined. Significance of differences in protein abundance and half-life between UPS-dependent and UPS-independent MHC class I peptide source proteins across all cell lines was determined by Fisher’s method combining *t* test *P* values from each cell line.

### Statistics

A limma test was performed for statistical significance between treatments for MHC class I peptide mass spectrometry experiments with R (*56*). Results were considered significant when the adjusted *P* value was <0.01. For statistical comparison of multiple treatments with vehicle, an analysis of variance (ANOVA) was performed; if there was a treatment effect significant at *P* < 0.05, Dunnett’s test was performed. When *t* tests were performed for multiple cell lines, Fisher’s method was used to determine a single meta-analysis *P* value. To calculate enrichment of categorical terms in a single cell line, Fisher’s exact test with Bonferroni multiple testing corrections was performed. To calculate enrichment of categorical terms across multiple cell lines, a Cochran-Mantel-Haenszel test with Bonferroni multiple testing corrections was performed. Bar graphs with error bars represent average + SEM.
