# Supplementary material for: Quantitative measurement of the requirement of diverse protein degradation pathways in MHC class I peptide presentation
Source: Sci Adv. 2023 Jun 23;9(25):eade7890. doi: 10.1126/sciadv.ade7890 (PMC10289651; doi:10.1126/sciadv.ade7890)
Supplement: Supplementary file 1 — Figs. S1 to S6 Legends for tables S1 to S11 [file sciadv.ade7890_sm.pdf]

Science Advances
AAAS

Supplementary Materials for

Quantitative measurement of the requirement of diverse protein degradation pathways in MHC class I peptide presentation

Jennifer L. Mamrosh *et al.*

Corresponding author: Raymond J. Deshaies, rdii2003@gmail.com

Sci. Adv. **9**, eade7890 (2023)
DOI: 10.1126/sciadv.ade7890

The PDF file includes:

Figs. S1 to S6
Legends for tables S1 to S11

Other Supplementary Material for this manuscript includes the following:

Tables S1 to S11

Supplementary Excel Files

Table S1. Detailed description of MHC Class I peptide mass spectrometry datasets

Table S2. MHC Class I peptide presentation after brefeldin A treatment to block MHC Class I secretion in HCC1954BL cells

Table S3. MHC Class I peptide presentation after acid stripping, brefeldin A treatment, or translation inhibition in HCC1954BL and HCC38BL cells

Table S4. MHC Class I peptide presentation after ubiquitin, proteasome, or translation inhibition in HCC1143BL, HCC1954BL, HCC1395BL, and HCC38BL cells

Table S5. MHC Class I peptide presentation after apoptosis-inducing agent cisplatin treatment in HCC1954BL cells

Table S6. MHC Class I peptide presentation after proteasome, autophagy, or translation inhibition in HCC1954BL cells

Table S7. MHC Class I peptide presentation after proteasome, TPPII, protease, or translation inhibition in HCC1954BL cells

Table S8. MHC Class I peptide presentation after proteasome, p97, or translation inhibition in HCC1954BL cells

Table S9. MHC Class I peptide presentation after Cullin-RING E3 ligase modulation or translation inhibition in HCC1954BL cells

Table S10. MHC Class I peptide presentation after partial proteasome inhibition in HCC1143BL, HCC1954BL, HCC1395BL, and HCC38BL cells

Table S11. MHC Class I peptide presentation after partial proteasome inhibition in HCC1954 breast cancer cells

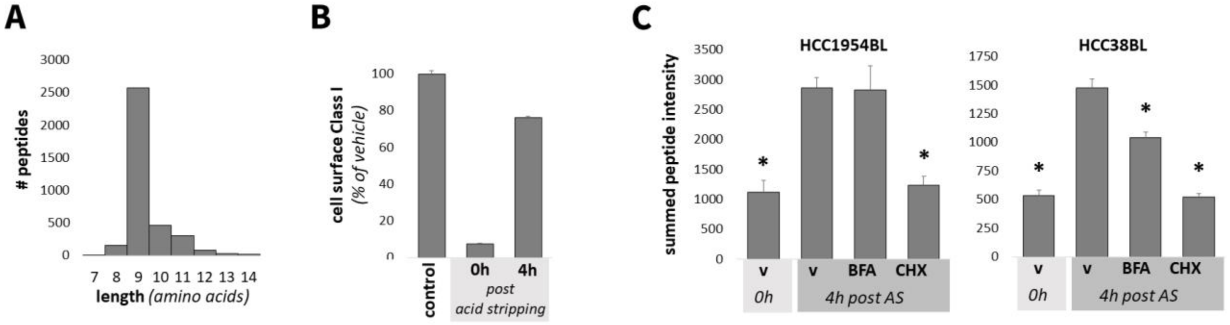

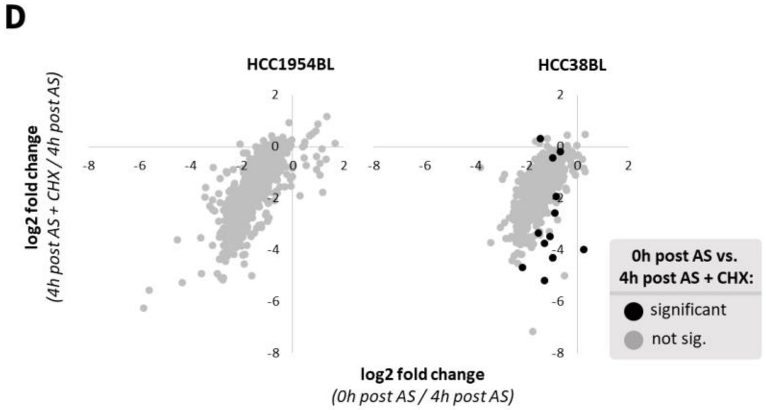

E

	HLA-A	*HLA-B*	*HLA-C*
**HCC1143BL**	*31:01 / 31:01*	*35:08 / 37:01*	*04:01 / 06:02*
**HCC1954BL**	*24:02 / 24:02*	*35:01 / 40:06*	*04:01 / 15:02*
**HCC1395BL**	*29:02 / 29:02*	*08:01 / 45:01*	*06:02 / 07:01*
**HCC38BL**	*03:01 / 03:01*	*35:03 / 35:03*	*12:03 / 12:03*

Supplementary Figure 1 (A) Histogram of MHC Class I peptide length for all peptides identified in HCC1954BL cells treated with vehicle or 5 µM Brefeldin A for 16h. **(B)** Flow cytometry measurement of cell surface MHC Class I for untreated HCC1954BL cells (control), those collected immediately following mild acid elution to remove MHC Class I peptides (0h post acid stripping), and those collected 4h after acid stripping (n=3/group). **(C)** Cells were pre-treated with vehicle, cycloheximide (25µg/ml), or Brefeldin A (5 µM) for 2h, and pre-existing MHC Class I peptides removed by mild acid elution ("acid stripping"; AS). Cells were immediately collected after AS, or treated again with vehicle, cycloheximide, or BFA for 4h. Summed peptide intensity quantified by mass spectrometry is depicted. * indicates $p < 0.01$, as compared with 4h post AS + vehicle, by Dunnett's test. **(D)** Scatterplots show the log2 fold change in displayed MHC Class I peptides from cells collected immediately after AS versus those collected 4h after

AS (x-axis) or from cells collected 4h after AS with cycloheximide treatment versus those collected 4h after AS (y-axis). Black indicates peptides significantly different between cells collected immediately after AS versus those collected 4h after AS with cycloheximide treatment. **(E)** MHC Class I alleles for *HLA-A*, *-B*, & *-C* genes for B lymphoblast cell lines used.

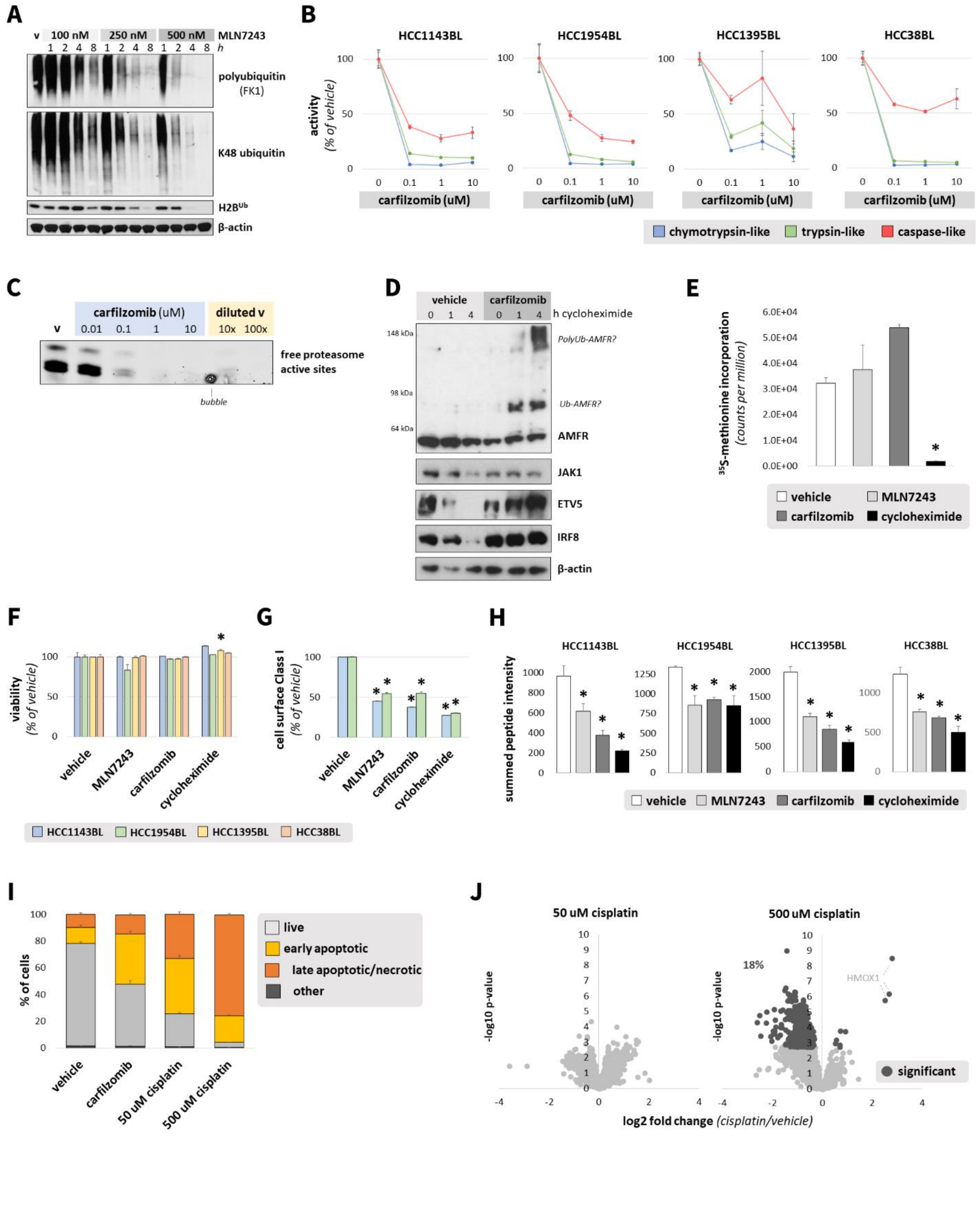

Supplementary Figure 2 (A) HCC1954BL cells were treated with doses of the E1 ubiquitination inhibitor MLN7243 or vehicle (v) as indicated, for increasing lengths of time. Polyubiquitin, K48-linked ubiquitin,

and ubiquitinated histone H2B (Lys120) were immunoblotted, with β-actin used as a loading control. **(B)** B lymphoblast cell lines were treated with doses of the proteasome inhibitor carfilzomib as indicated for 1h. Activity of the chymotrypsin-like, trypsin-like, and caspase-like sites of the proteasome was measured using fluorescent substrates (Suc-LLVY-AMC, Boc-LRR-AMC, and Ac-nLPnLD-AMC, respectively) incubated with cell lysates for 1h at 37°C. Percent inhibition was calculated relative to vehicle treated cells (n=3/group). **(C)** HCC1954BL cells were treated with vehicle (v) or increasing doses of carfilzomib for 1h. Free proteasome active sites were then labeled with 500 nM Me4BodipyFL-Ahx3Leu3VS for 1h. Cells were lysed and equivalent protein amounts (excluding rightmost two lanes) were loaded onto Tricine SDS-PAGE gels to visualize free proteasome subunits. Vehicle treated cells were also diluted 1:10 and 1:100 in lysis buffer, to represent 90% and 99% loss of free proteasome active sites, respectively. **(D)** HCC1954BL cells were treated with vehicle or 1 µM carfilzomib for 5 min. Cells were then treated with 25 µg/ml cycloheximide to inhibit translation and collected at timepoints listed. Equivalent protein amounts were loaded onto SDS-PAGE gels and immunoblotted for known ubiquitin-proteasome system substrates with reported short half lives in B cells. B-actin was used as a loading control. **(E)** HCC1954BL cells were treated with vehicle, 500 nM MLN7243 (4h), 1 µM carfilzomib (1h), or 25 µg/ml cycloheximide (2h) (n=3/group). In the final 30 min of treatment, methionine-deficient media was used. Newly synthesized proteins were then labeled with [^{35}S]methionine for 5 min, and incorporation of radiolabeled methionine measured in TCA precipitated proteins. * indicates $p < 0.05$, as compared with vehicle, by Dunnett's test. **(F)** Viability of cells collected for mass spectrometry as measured by trypan blue staining, relative to viability of vehicle treated cells, for cells treated with MLN7243 (500 nM; 4h pretreatment), carfilzomib (1 µM; 1h pretreatment), and cycloheximide (25 µg/ml; 2h pretreatment) for 4h following acid stripping. * indicates $p < 0.01$ versus vehicle by Dunnett's test. **(G)** Flow cytometry measurement of cell surface MHC Class I for HCC1954BL cells treated with MLN7243 (500 nM; 4h pretreatment), carfilzomib (1 µM; 1h pretreatment), and cycloheximide (25

µg/ml; 2h pretreatment) (n = 3/group) for 4h following acid stripping. * indicates $p < 0.01$ versus vehicle by Dunnett's test. **(H)** Summed peptide intensity quantified by mass spectrometry for cells treated as described in Supplementary Figure 2F. * indicates $p < 0.01$ versus vehicle by Dunnett's test. **(I)** Percent of HCC1954BL cells considered early apoptotic, late apoptotic/necrotic, live (not apoptotic or necrotic), or unclassifiable ("other") after the following treatments for 4h post acid stripping: vehicle, 1 µM carfilzomib (1h pre-treatment), 50 µM cisplatin (20h pre-treatment), or 500 µM cisplatin (20h pre-treatment) (n=3/group). Staining for Annexin V was considered indicative of apoptosis and staining for propidium iodide indicative of necrosis. **(J)** Volcano plots representing quantitative changes in MHC Class I peptide presentation upon cisplatin treatment. Cells were treated for 4h post acid stripping with the following treatments: vehicle, 50 µM cisplatin (20h pre-treatment), and 500 µM cisplatin (20h pre-treatment).

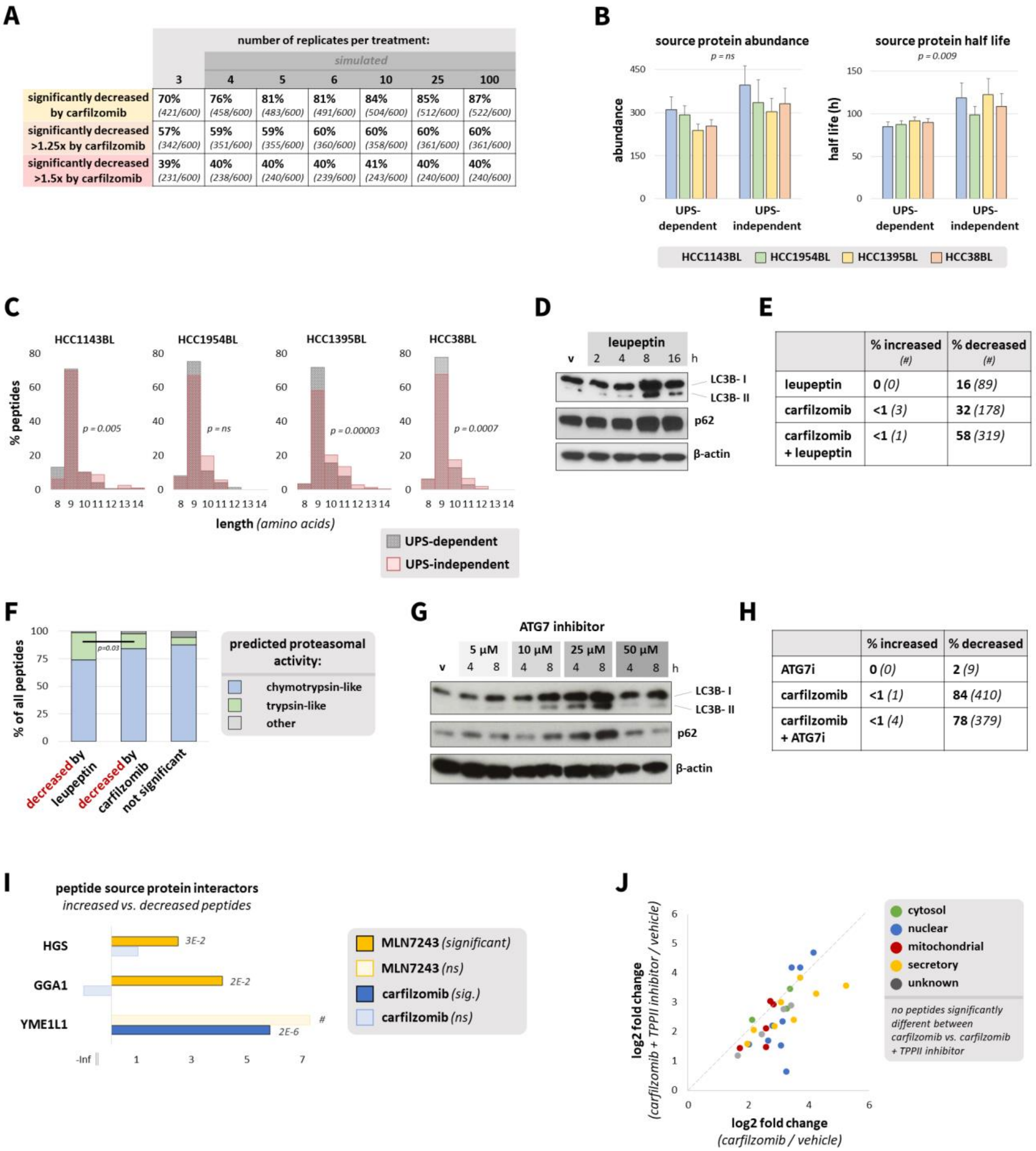

Supplementary Figure 3 (A) HCC1954BL cells were treated with MLN7243 (500 nM; 4h pretreatment), carfilzomib (1 µM; 1h pretreatment), and cycloheximide (25 µg/ml; 2h pretreatment) for 4h following acid stripping. Simulated replicates were generated from a Gaussian distribution specific to each peptide and treatment, and significance was determined by limma test. **(B)** In each cell line, source protein

abundance and half-life for peptides not significantly decreased by MLN7243 and carfilzomib ("UPS-independent") versus those significantly decreased by MLN7243 and carfilzomib ("UPS-dependent"). Significance was determined by Fisher's method. **(C)** Cells were treated with MLN7243 (500 nM; 4h pretreatment), carfilzomib (1 μM; 1h pretreatment), or cycloheximide (25 μg/ml; 2h pretreatment) for 4h following acid stripping. Peptides not decreasing more than 1.5 fold in response to cycloheximide were excluded. Histogram depicts peptide length for peptides not significantly decreased by MLN7243 and carfilzomib ("UPS-independent") versus those significantly decreased by MLN7243 and carfilzomib ("UPS-dependent"). **(D)** HCC1954BL cells were treated with 50 μM leupeptin for timepoints indicated, and lysates were immunoblotted for LC3B, p62, and loading control β-actin. **(E)** HCC1954BL cells were treated for 4h with carfilzomib (1 μM; 1h pretreatment) and/or leupeptin (50 μM; 1h pretreatment); peptides not decreasing more than 1.5 fold in response to 25 μg/ml cycloheximide were excluded. Chart indicates percent (bold) and number (italicized) of MHC Class I peptides significantly increased or decreased by each treatment. **(F)** MHC Class I peptides significantly decreased in response to leupeptin treatment or carfilzomib treatment, and those peptides not significantly changed, were characterized as one of the following: likely to be produced by proteasomal chymotrypsin-like activity, likely to be produced by trypsin-like activity, and likely to be produced by other proteolytic activity. The significance of the fraction of peptides likely to be produced by trypsin-like activity was assessed by Fisher's exact test between the following groups: peptides decreased by leupeptin treatment and peptides decreased by carfilzomib treatment. **(G)** HCC1954BL cells were treated with 5, 10, and 25 μM ATG7 inhibitor for timepoints indicated, and lysates were immunoblotted for LC3B, p62, and loading control β-actin. **(H)** HCC1954BL cells were treated for 4h with carfilzomib (1 μM; 1h pretreatment) and/or ATG7 inhibitor (25 μM; 1h pretreatment); peptides not decreasing more than 1.5 fold in response to 25 μg/ml cycloheximide were excluded. Chart indicates percent (bold) and number (italicized) of MHC Class I peptides significantly increased or decreased by each treatment. **(I)** Enrichment of proteins interacting

with source proteins for MHC Class I peptides significantly increased versus peptides significantly decreased in cells treated with MLN7243 or carfilzomib. Cells were treated as in Supplementary Figure 3C. Protein interactors of source proteins for peptides significantly increased and peptides significantly decreased by MLN7243 and carfilzomib were obtained from BioGRID. A Cochran–Mantel–Haenszel test was used to test the enrichment of protein interactions across all cell lines; significant adjusted p-values are reported. “#” indicates insufficient interactions to calculate significance. ”-Inf” reflects no interactions in the increased peptide group. **(J)** HCC1954BL cells were treated for 4h with carfilzomib (1 μM; 1h pretreatment) and/or an inhibitor of tripeptidyl peptidase II (20 μM AAF-CMK; 1h pretreatment). Only MHC Class I peptides significantly increased by carfilzomib are depicted. Scatterplots depict the log2 fold change of peptides for the following comparisons: carfilzomib versus vehicle (x-axis) and carfilzomib & a TPPII inhibitor versus vehicle (y-axis).

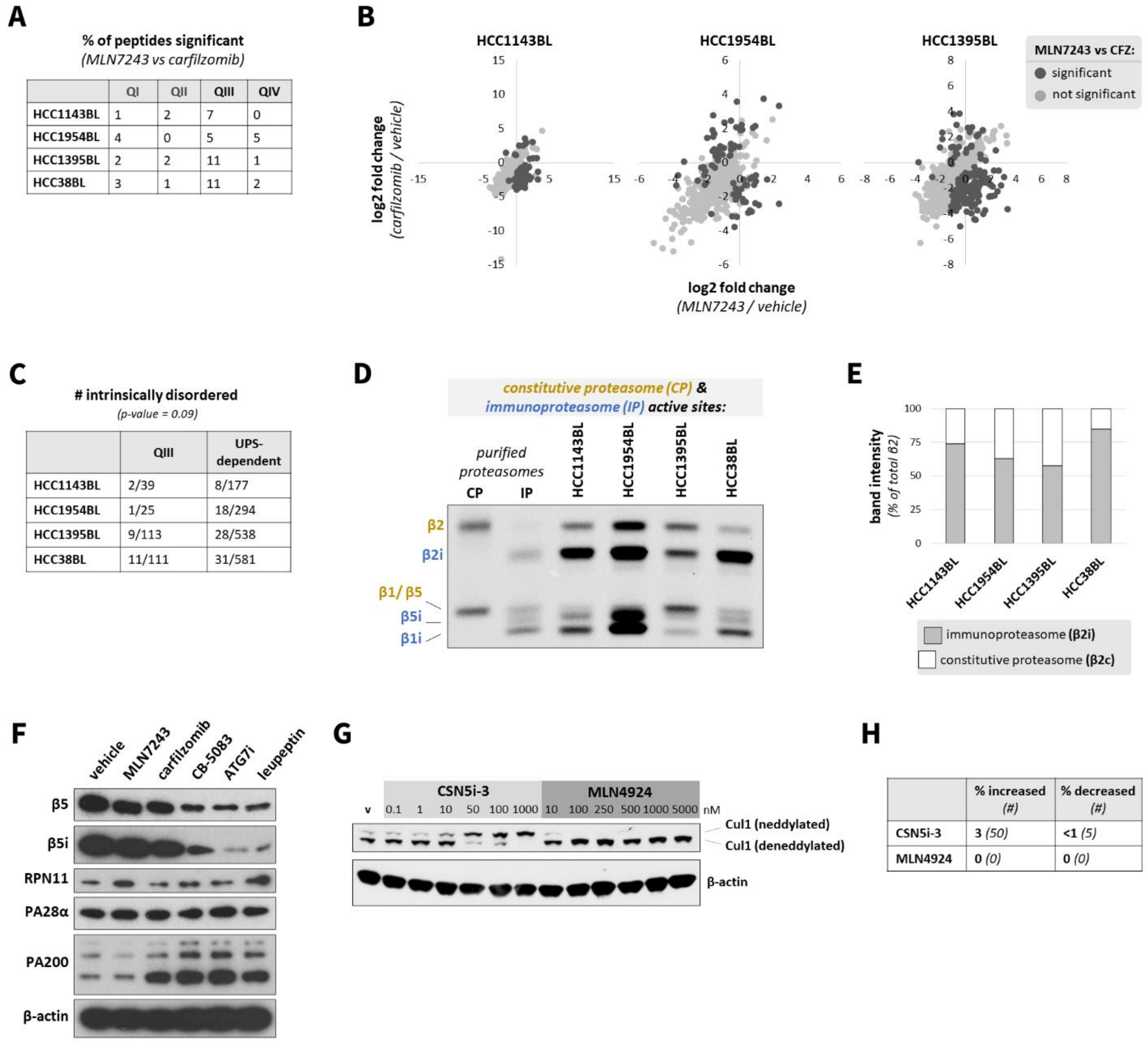

Supplementary Figure 4 (A) Cells were treated for 4h with vehicle, MLN7243 (500 nM; 4h pretreatment), or carfilzomib (1 µM; 1h pretreatment). MHC Class I peptides not decreasing more than 1.5 fold in response to 25 µg/ml cycloheximide were excluded. Peptides significantly different between MLN7243 and carfilzomib treatment were assigned to “quadrants”, as depicted in Figure 3A. Table displays percent of peptides in each quadrant by cell line. **(B)** Cells were treated as in Supplementary Figure 3A. Scatterplot depicts the log2 fold change of MHC Class I peptides for the following

comparisons: MLN7243 versus vehicle (x-axis), and carfilzomib versus vehicle (y-axis). **(C)** Number of proteins in QIII and "UPS-dependent" (significantly reduced by MLN7243 and CFZ) considered "intrinsically disordered" as classified by DisProt. A Cochran–Mantel–Haenszel test was used to determine whether the fraction of peptides considered intrinsically disordered across cell lines differed between the QIII and UPS-dependent groups. **(D)** B lymphoblast cell lines were treated with 500 nM Me4BodipyFL-Ahx3Leu3VS for 1h to label proteasome active sites. Equivalent protein amounts were loaded onto Tricine SDS-PAGE gels to resolve constitutive proteasome (yellow) and immunoproteasome (blue) subunits. Purified constitutive proteasome (CP) and immunoproteasome (IP) were also analyzed. **(E)** Quantification of proteasome active site fluorescent gel band intensities as a measure of immunoproteasome to constitutive proteasome ratios in each cell line. Intensities of β2 (constitutive proteasome trypsin-like site) and β2i (immunoproteasome trypsin-like site) bands were calculated from the gel in Supplementary Figure 4D. Intensity ratios are depicted. **(F)** HCC1954BL cells were treated with vehicle or the following inhibitors: MLN7243 (500 nM; 8h), carfilzomib (1 µM; 5h), CB-5083 (5 µM; 5h), an ATG7 inhibitor (25 µM; 5h), and leupeptin (50 µM; 5h). Lysates were immunoblotted for constitutive proteasome active site β5, immunoproteasome active site β5i, 19S deubiquitinating enzyme RPN11, alternative proteasome cap subunit PA28α, alternative proteasome cap PA200, and loading control β-actin. **(G)** HCC1954BL cells were treated with increasing doses of neddylation inhibitor MLN4924 or COP9 signalosome inhibitor CSN5i-3 for 2h. Lysates were immunoblotted for neddylated/deneddylated CUL1, with β-actin used as a loading control. **(H)** HCC1954BL cells were treated for 4h with vehicle, MLN4924 (250 nM; 2h pretreatment), or CSN5i-3 (1 µM; 2h pretreatment). MHC Class I peptides not decreasing more than 1.5 fold in response to 25 µg/ml cycloheximide were excluded. Chart represents percent (bold) and number (italicized) of peptides significantly increasing and decreasing by MLN4924 and CSN5i-3 treatment.

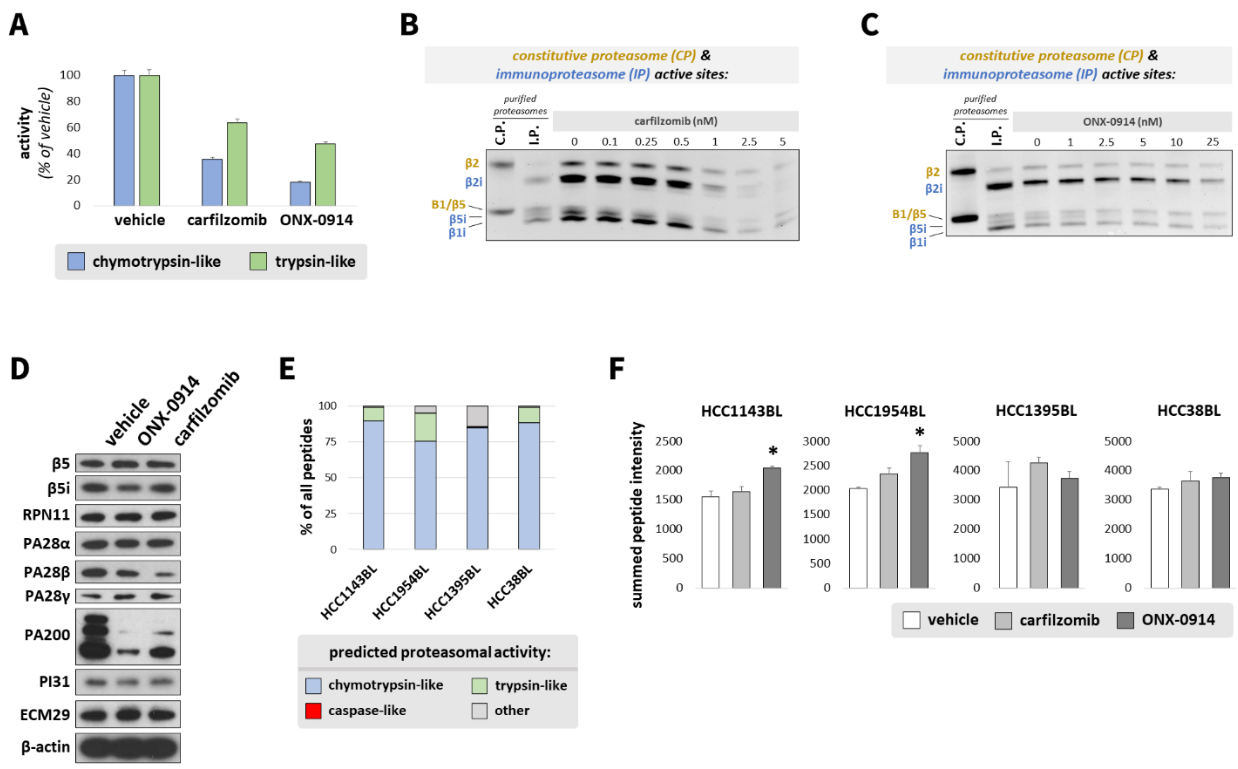

G

carfilzomib

	% increased (#)	% decreased (#)
HCC1143BL	9 *(219)*	4 *(100)*
HCC1954BL	24 *(843)*	7 *(253)*
HCC1395BL	4 *(117)*	0 *(0)*
HCC38BL	0 *(6)*	0 *(1)*

ONX-0914

	% increased (#)	% decreased (#)
HCC1143BL	37 *(876)*	9 *(210)*
HCC1954BL	42 *(1453)*	8 *(295)*
HCC1395BL	3 *(76)*	0 *(0)*
HCC38BL	20 *(928)*	6 *(281)*

H

	increased peptides: trypsin- like activity	decreased peptides: trypsin- like activity	p-value	odds ratio
HCC1143BL	90/875	7/211	7E-4	3.3
HCC1954BL	323/1453	37/295	1E-4	2.0
HCC1395BL	1/76	0/0	-	-
HCC38BL	151/928	26/281	3E-3	1.9

Supplementary Figure 5 (A) HCC1954BL cells were treated for 48h with vehicle, carfilzomib (5 nM), or immunoproteasome inhibitor ONX-0914 (25 nM) to inhibit the chymotrypsin-like site of the proteasome. Activity of the chymotrypsin-like and trypsin-like sites of the proteasome was measured using fluorescent substrates (Suc-LLVY-AMC and Boc-LRR-AMC, respectively) incubated with cell lysates for 1h at 37°C. Percent inhibition was calculated relative to vehicle treated cells. **(B,C)** HCC1954BL cells were treated with increasing doses of carfilzomib or ONX-0914 for 48h, and 500 nM Me4BodipyFL-Ahx3Leu3VS was added for the final 1h to label proteasome active sites. Equivalent protein amounts

were loaded onto Tricine SDS-PAGE gels to resolve constitutive proteasome (yellow) and immunoproteasome (blue) subunits. Purified constitutive proteasome (CP) and immunoproteasome (IP) were also analyzed. **(D)** HCC1954BL cells were treated with vehicle, carfilzomib (5 nM), or ONX-0914 (25 nM) for 48h. Lysates were immunoblotted for constitutive proteasome active site β5, immunoproteasome active site β5i, 19S deubiquitinating enzyme RPN11, alternative proteasome cap subunits PA28α, PA28β, and PA28γ, alternative proteasome caps PA200, PA3I, and ECM29, and loading control β-actin. **(E)** All peptides in B lymphoblast cell lines were characterized as one of the following: likely to be produced by proteasomal chymotrypsin-like activity, likely to be produced by proteasomal trypsin-like activity, likely to be produced by proteasomal caspase-like activity, and likely to be produced by other proteolytic activity. **(F)** Cells were treated with vehicle, carfilzomib (5 nM), or ONX-0914 (25 nM) for 48h. Graph depicts summed MHC Class I peptide intensity; * indicates $p < 0.01$ by Dunnett's test. **(G)** Percent (bold) and number (italicized) of MHC Class I peptides significantly increased or decreased by carfilzomib or ONX-0914 treatment across cell lines. **(H)** The number of MHC Class I peptides considered "trypsin activity-like" (containing a C-terminal lysine or arginine) was determined for peptides significantly increased and decreased by ONX-0914 treatment versus vehicle. Significance was determined by Fisher's exact test.

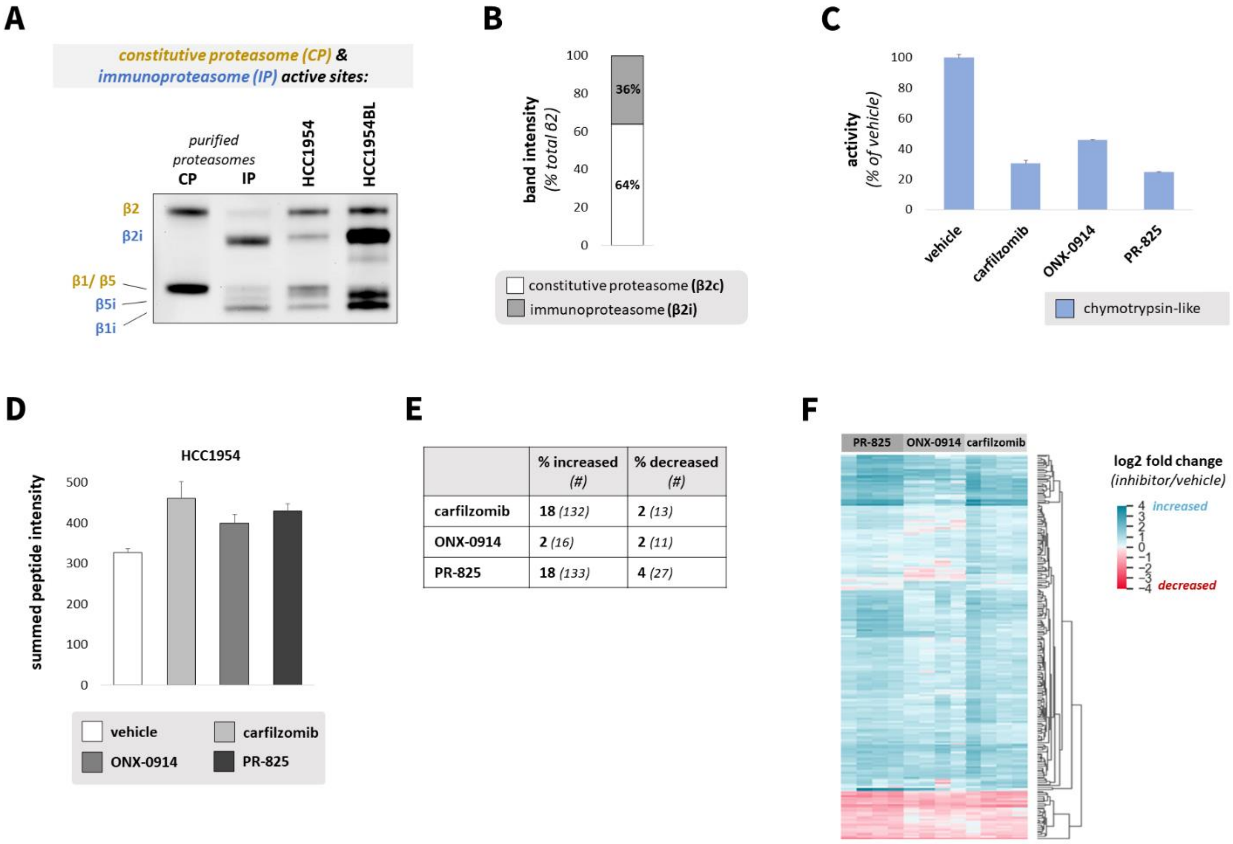

Supplementary Figure 6 (A) HCC1954 and HCC1954BL cells were treated with 500 nM Me4BodipyFL-Ahx3Leu3VS for 1h to label proteasome active sites. Equivalent protein amounts were loaded onto Tricine SDS-PAGE gels to resolve constitutive proteasome (yellow) and immunoproteasome (blue) subunits. Purified constitutive proteasome (CP) and immunoproteasome (IP) were also analyzed. **(B)** Quantification of proteasome active site fluorescent gel band intensities as a measure of immunoproteasome to constitutive proteasome ratios in HCC1954 cells. Intensities of β2 (constitutive proteasome trypsin-like site) and β2i (immunoproteasome trypsin-like site) bands were calculated from the gel in Supplementary Figure 5E. Intensity ratios are depicted. **(C)** HCC1954 cells were treated with vehicle, 75 nM carfilzomib, 200 nM ONX-0914, or 250 nM constitutive proteasome inhibitor PR-825 for 48h. Activity of the chymotrypsin-like site of the proteasome was measured using a fluorescent substrate (Suc-LLVY-AMC) incubated with cell lysates for 1h at 37°C. Percent inhibition was calculated

relative to vehicle treated cells (n=3/group). **(D)** HCC1954 cells were treated with vehicle, carfilzomib (75 nM), ONX-0914 (200 nM), or PR-825 (250 nM) for 48h. Graph depicts summed MHC Class I peptide intensity. **(E)** Percent (bold) and number (italicized) of MHC Class I antigens significantly increased or decreased by carfilzomib, ONX-0914, or PR-825 in HCC1954 cells is listed. **(F)** Heat map shows log2 fold change (proteasome inhibitor/vehicle) for MHC Class I peptides in HCC1954 cells for peptides significant in at least one treatment.
